# Structural Mechanisms of Store-Operated and Mitochondrial Calcium Regulation: Initiation Points for Drug Discovery

**DOI:** 10.3390/ijms21103642

**Published:** 2020-05-21

**Authors:** Megan Noble, Qi-Tong Lin, Christian Sirko, Jacob A. Houpt, Matthew J. Novello, Peter B. Stathopulos

**Affiliations:** 1 Department of Physiology and Pharmacology, Schulich School of Medicine and Dentistry, The University of Western Ontario, London, ON N6A5C1, Canada; mnoble29@uwo.ca (M.N.); qlin44@uwo.ca (Q.-T.L.); csirko@uwo.ca (C.S.); mnovell@uwo.ca (M.J.N.); 2 Department of Medicine, Schulich School of Medicine and Dentistry, The University of Western Ontario, London, ON N6A5C1, Canada; jhoupt2022@meds.uwo.ca

**Keywords:** calcium signaling, store-operated calcium entry, stromal interaction molecule, Orai1, mitochondrial calcium uniporter, leucine-zipper EF-hand containing transmembrane protein-1, ryanodine receptor, inositol 1,4,5 trisphosphate receptor, protein structure, drug development

## Abstract

Calcium (Ca^2+^) is a universal signaling ion that is essential for the life and death processes of all eukaryotes. In humans, numerous cell stimulation pathways lead to the mobilization of sarco/endoplasmic reticulum (S/ER) stored Ca^2+^, resulting in the propagation of Ca^2+^ signals through the activation of processes, such as store-operated Ca^2+^ entry (SOCE). SOCE provides a sustained Ca^2+^ entry into the cytosol; moreover, the uptake of SOCE-mediated Ca^2+^ by mitochondria can shape cytosolic Ca^2+^ signals, function as a feedback signal for the SOCE molecular machinery, and drive numerous mitochondrial processes, including adenosine triphosphate (ATP) production and distinct cell death pathways. In recent years, tremendous progress has been made in identifying the proteins mediating these signaling pathways and elucidating molecular structures, invaluable for understanding the underlying mechanisms of function. Nevertheless, there remains a disconnect between using this accumulating protein structural knowledge and the design of new research tools and therapies. In this review, we provide an overview of the Ca^2+^ signaling pathways that are involved in mediating S/ER stored Ca^2+^ release, SOCE, and mitochondrial Ca^2+^ uptake, as well as pinpoint multiple levels of crosstalk between these pathways. Further, we highlight the significant protein structures elucidated in recent years controlling these Ca^2+^ signaling pathways. Finally, we describe a simple strategy that aimed at applying the protein structural data to initiating drug design.

## 1. Cytosolic and Stored Calcium

Regulating cytosolic calcium ion (Ca^2+^) levels is fundamental for cellular life. Under resting conditions, intracellular Ca^2+^ concentrations are maintained at ~100 nM by effectively segregating Ca^2+^ into the extracellular fluid and within intracellular stores, such as the mitochondria and sarco/endoplasmic reticulum (S/ER). Under basal conditions, Ca^2+^ levels within the cytosol and mitochondria are similar; conversely, in times of cellular stress or injury, mitochondria can uptake Ca^2+^ [1,2,3,4] in order to prevent cytosolic Ca^2+^ overload [5,6,7]. Persistently high levels of mitochondrial Ca^2+^ would lead to a cycle of higher respiratory chain activity and the generation of reactive oxygen species (ROS), resulting in oxidative stress, apoptosis, and/or necrosis [8,9]. Ca^2+^ is a cofactor that is required for mitochondrial dehydrogenase function, which are enzymes integral to the electron transport chain [10,11]. The primary mode of mitochondrial Ca^2+^ uptake is via the low affinity, high capacity mitochondrial Ca^2+^ uniporter (MCU) complex [1,12,13]. However, recent evidence suggests that the bi-directional leucine-zipper EF-hand containing transmembrane protein-1 (LETM1) Ca^2+^/proton (H^+^) antiporter is involved in fine-tuning these levels [14,15]. Thus, the MCU complex and LETM1 are emerging as two critical regulators of mitochondrial Ca^2+^ uptake, dictating both mitochondrial and cellular function.

The S/ER is the largest intracellular Ca^2+^ store, maintaining free Ca^2+^ in the ~400–700 µM range (depending on cell-type) [16,17,18,19,20,21], which can be released into the cytosol in order to mediate and/or trigger a multitude of downstream cellular processes [22]. In excitable and non-excitable cells, ER-membrane bound ryanodine receptors (RyRs) and inositol 1,4,5-trisphosphate receptors (IP_3_Rs), respectively, serve as the predominant channels that release Ca^2+^ into the cytosol from the S/ER stores [23,24,25]. A subsequent entry of extracellular Ca^2+^ is critical for the activation of Ca^2+^-dependent signaling cascades that require more chronic elevations in cytosolic Ca^2+^ because S/ER Ca^2+^ stores are limited in capacity. Store-operated Ca^2+^ entry (SOCE) is the process whereby S/ER Ca^2+^ store depletion leads to the activation of plasma membrane (PM) Ca^2+^ channels for a sustained Ca^2+^ entry into the cytosol from the extracellular space [26,27,28,29]. The principal molecular machinery mediating SOCE include the Ca^2+^-sensing S/ER-membrane inserted stromal interaction molecules (STIMs) [30,31] and the pore-forming Orai1 channels on the PM [32,33,34,35,36]. The elevated cytosolic Ca^2+^ levels that are driven by SOCE not only signal downstream physiological and pathophysiological cellular responses, but also replenish the depleted intracellular Ca^2+^ stores.

### 1.1. Mobilization of S/ER Calcium Stores

Across human cell types, several mechanisms of extracellular signaling exist, which ultimately culminate in the stimulation or sensitization of IP_3_Rs and/or RyRs, leading to the depletion of the S/ER Ca^2+^ stores [23,24,37]. Such extracellular signaling pathways can involve G-protein-coupled receptors (GPCRs), receptor tyrosine kinases (RTKs), T- and B-cell receptors (TCRs, BCRs), or voltage-gated ion channels (VGICs), presenting a variety of mechanisms for S/ER Ca^2+^ store release (Table 1), which activate Ca^2+^-dependent transcription factors, structural proteins, and enzymes that are critical for cellular function.

GPCRs constitute a major family of PM receptors that, when activated by the appropriate extracellular ligand, can serve to mediate the release of Ca^2+^ from the S/ER into the cytosol via IP_3_Rs [37,42]. Upon extracellular ligand binding-induced activation, GPCRs activate intracellular phospholipases, such as Cβ or Cγ, which cleave phosphatidylinositol 4,5-bisphosphate into diacylglycerol and inositol 1,4,5-trisphosphate (IP_3_) [42]. IP_3_ is a small diffusible second messenger that binds and activates IP_3_Rs, causing S/ER Ca^2+^ store release into the cytosol [65]. This IP_3_R-driven Ca^2+^ signaling mechanism is critical in platelets where thrombin binding to protease activated receptor (PAR)-1 or PAR4 mediates thrombosis [41], in vascular endothelial cells where bradykinin binding to the bradykinin receptors (BR)-1 or BR2 causes vasodilation [66], and in airway smooth muscle cells where histamine binding to the histamine H_1_ receptor (H_1_R) leads to bronchoconstriction [67], to name a few examples.

Nevertheless, it is not only GPCRs that can induce S/ER Ca^2+^ release through IP_3_R activation. RTKs, such as insulin growth factor-1 receptor in skeletal myoblasts activate phospholipase C and IP_3_ production upon binding insulin-like growth factor-1, leading to ER Ca^2+^ release necessary for differentiation in myogenesis [68,69]. In similar mechanisms, when the membrane immunoglobulin (Ig) component of a BCR, such as IgM-BCR, interacts with an antigen or when T-cells bind self-antigen presented by thymic epithelial cells via TCRs, phosphorylation cascades result in phospholipase C and IP_3_R activation, enabling humoral immunity signaling and apoptosis/elimination of self-reactive T-cells, respectively [51,70]. Likewise, this TCR signaling pathway is vital to mediating immune responses after interacting with foreign antigens [16,17]. Immunologically, antigen-antibody complexes can also bind to different types of Fc receptors on mast cells, macrophages, dendritic cells or natural killer cells, inducing phospholipase C-mediated IP_3_ generation and S/ER Ca^2+^ release [16,17,55,56].

RyRs are much larger structural homologues of IP_3_Rs and similarly function to release S/ER Ca^2+^ [24]. For example, in cardiomyocytes, VGICs on the PM (i.e., L-type Ca^2+^ channels) open in response to membrane depolarization, resulting in the entry of Ca^2+^ into the cytosol from the extracellular space [60,61]. This cytosol entered Ca^2+^ binds to S/ER RyRs, opening these channels, in contrast to IP_3_Rs, which require IP_3_. Remarkably, the RyR-mediated release of S/ER Ca^2+^ can propagate to adjacent channels and promote additional RyR activation in a process that is known as Ca^2+^-induced Ca^2+^ release [60,61].

### 1.2. Store-Operated Calcium Entry

**Figure 1:**
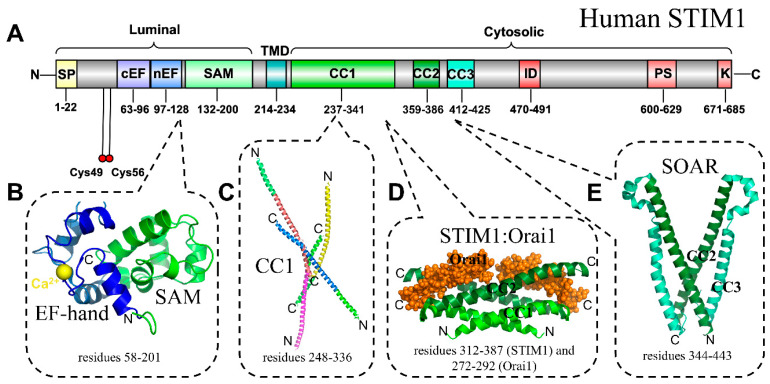
Structural elucidation of individual human STIM1 domains. (**A**) Domain architecture of human STIM1 [National Center for Biotechnology Information (NCBI) accession AFZ76986]. The highly conserved domains and regions with well-defined functional roles are indicated with residue ranges shown below the respective segment. The residue ranges for EF-SAM and the coiled-coils are from the solution structure and Coils analysis (using a 0.1 probability cutoff and 14, 21, and 28 residue combination) [105], respectively. All other residue range annotations are from Uniprot accession Q13586. The location of the luminal Cys residues that undergo post-translation modifications to modify Ca^2+^-sensing function are indicated (red spheres). The topological orientation relative to the S/ER is indicated above the diagram. (**B**) Solution NMR structure of Ca^2+^ loaded STIM1 EF-SAM. (**C**) Cluster of four CC1 monomers showing several different interfaces revealed by STIM1 CC1 crystallization. (**D**) Solution structure of the CC1-CC2 dimer in complex with two Orai1 C-terminal peptides (orange spheres). (**E**) Crystal structure of the dimerized STIM1-Orai activation region (SOAR). In **B**–**E**, colours are shaded, as depicted in (**A**), and overlapping coiled-coil region colours are preserved between structures. The pdb coordinate files for STIM1 EF-SAM, CC1, CC1-CC2:Orai1, and SOAR are from 2K60 [75], 4O9B [106], 2MAK [107], and 3TEQ [108], respectively. SP, signal peptide; cEF, canonical EF-hand; nEF, non-canonical EF-hand; SAM, sterile alpha motif; TMD, transmembrane domain; CC1 -2 -3, coiled-coil -1 -2 -3; ID, inhibitory domain; PS, pro/ser-rich region; K, Lys-rich (polybasic) region; N, amino terminus; C, carboxyl terminus.

**Figure 2:**
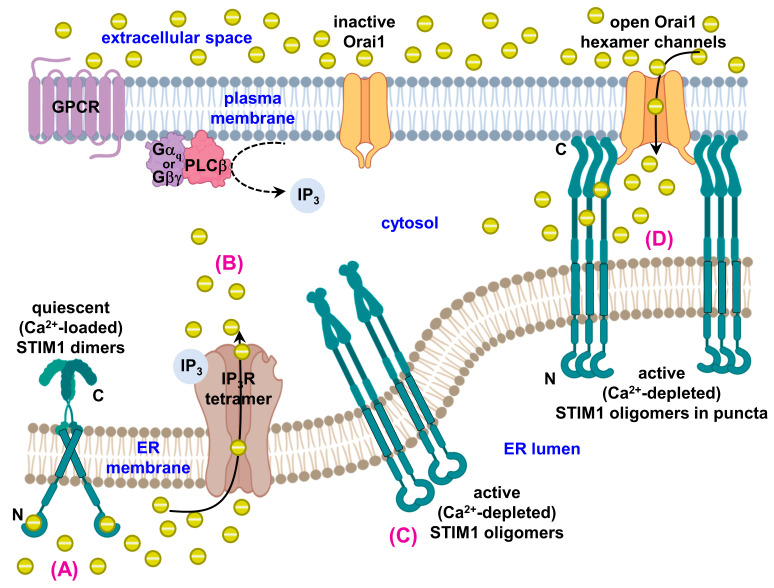
Molecular mechanism of store-operated Ca^2+^ entry (SOCE) signaling. (**A**) With replete S/ER luminal Ca^2+^ stores, STIM1 (green cartoon) adopts a quiescent conformation. Quiescent STIM1 is dimerized via interactions between cytosolic domains [108,109,110] and remains in a compact conformation due to Ca^2+^ (yellow spheres) binding by EF-SAM, which keeps these luminal domains in monomeric states [72,73,75]. (**B**) Upon cell stimulation, via G protein-mediated PLC activation for example, IP_3_ is generated and diffuses to the IP_3_Rs (brown cartoon) on the S/ER membrane. IP_3_ binding to IP_3_Rs, opens these receptor Ca^2+^ channels, depleting the S/ER Ca^2+^ stores [28,111,112]. (**C**) Ca^2+^ unbinding from EF-SAM causes luminal domain dimerization [73,75,113], which rearranges the transmembrane regions [114] and promotes coiled-coil-1 interactions, culminating in a conformational extension of the STIM1 cytosolic region [74,76,115]. This structural change also promotes further oligomerization of STIMs via the cytosolic domains [110,115]. (**D**) Extended/oligomerized STIM1 gets trapped at S/ER-PM junctions [18,77,78,80] via interactions between the polybasic C-terminal regions and PM [78,95,116,117] as well as interactions between the STIM1 SOAR/CAD and Orai1 (orange cartoon) [82,83,107,118]. The direct interactions between STIM1 and Orai1 gate Orai1-composed hexameric Ca^2+^ release-activated Ca^2+^ (CRAC) channels [119,120], allowing Ca^2+^ to move from the extracellular space into the cytosol in SOCE. GPCR, G-protein coupled receptor; Gα_q_, G-alpha subunit; Gβγ, G-beta-gamma subunits; PLCβ, phospholipase Cβ; IP_3_, inositol trisphosphate; IP_3_R, IP_3_ receptor; STIM1, stromal interaction molecule-1; N, amino terminus; C, carboxyl terminus. This figure and the organelles depicted in the graphical abstract were created with BioRender.com.

SOCE is a ubiquitous Ca^2+^ signaling pathway that has evolved to use S/ER Ca^2+^ depletion as an activation signal, facilitating further and more sustained extracellular Ca^2+^ influx in response to the release of intracellular S/ER Ca^2+^ [26,27,28,29]. Stromal interaction molecule-1 (STIM1) is a single pass, type 1 transmembrane protein of 685 amino acids and the S/ER luminal Ca^2+^ sensor and SOCE activator [30,31] (Figure 1A). The luminal amino (N)-terminal region of STIM1 contains an ER signal peptide, EF-hand domain, and a sterile-alpha motif (SAM) domain. Included in the cytosolic segment of STIM1 is a long stretch of coiled-coil domains, a Pro-Ser-rich region, and a polybasic tail [71]. The EF-hand, together with the SAM domain, sense S/ER Ca^2+^ changes (Figure 1B–E). At resting S/ER Ca^2+^ concentrations, EF-SAM is bound to Ca^2+^ and in a quiescent monomeric state [72,73]. Upon the dissociation of Ca^2+^ from EF-SAM, destabilization and dimerization of this luminal domain occurs, causing conformational changes that propagate through to the cytosolic region of the molecule [74,75,76]. These Ca^2+^-depletion-induced structural changes lead to STIM1 oligomerization and trapping at S/ER-PM junctions [77,78,79,80]. At these junctions, a region of the STIM1 coiled-coil domains directly interacts with and gates Orai1 protein-composed Ca^2+^ channels on the PM, permitting Ca^2+^ influx into the cytosol from the extracellular space [81,82,83,84] (Figure 2).

A process known as Ca^2+^-dependent inactivation (CDI) deactivates Orai1 channels to terminate SOCE [85,86]. Multiple sites on STIM1 and Orai1 have been suggested to provide a negative feedback signal to SOCE, both dependent and independent of calmodulin function [87,88,89,90]. STIMs, like many proteins that are involved in mediating signaling cascades, are often subject to post-translational modifications, which affect both structure and function [27,91,92,93,94,95,96,97,98,99,100,101,102,103]. *S-*Glutathionylation, for example, is the covalent attachment of a glutathione (GSH) moiety to free cysteine (Cys) thiols. GSH modification of STIM1 at Cys56 has been shown to evoke constitutive Orai1 channel activation and Ca^2+^ entry independent of luminal Ca^2+^ concentrations [104]. It is worth speculating that the thiols are vital regulatory sites evolved for receiving distinct inputs depending on specific environmental cues given the high conservation of the luminal STIM Cys residues among vertebrates (Figure 1A).

### 1.3. STIM1, Orai1 and Disease

Several heritable mutations in STIM1 and Orai1 cause disease. The loss of function mutations in STIM1 occur as frameshift (i.e., E128RfsX9 [121]) and intronic (i.e., NM_003156.3:c.1538-1G>A [122]) variations leading to protein abrogation. Missense mutations exist that do not eliminate STIM1 protein (i.e., R429C [123] and P165Q [124]), but similarly lead to a loss of STIM1 function [125]. Orai1 loss of function mutations include missense (i.e., R91W [32] and A103E/L194P [126]) and frameshift (i.e., A88SfsX25 [126] and H165PfsX1 [127]), which can abrogate protein expression or disrupt the function of fully expressed protein. STIM1 and Orai1 loss of function mutations both lead to CRAC channelopathy syndrome. CRAC channelopathies are characterized by combined immunodeficiency with chronic infections in the central nervous system, respiratory tract, and gastrointestinal tracts, often leading to death. These channelopathies also present autoimmune hemolytic anemia and thrombocytopenia, muscular hypotonia and atrophy, anhidrosis, and defects in dental enamel development (reviewed in [128,129]). CRAC channelopathy due to STIM1 or Orai1 mutations is autosomal recessive.

Gain of function mutations occur in STIM1 (i.e., R304W [130], I115F [129], D84G, H109N, H109R, H72Q [131], N180T, L96V, F108I, F108L [132], and G81D [133]) and Orai1 (i.e., P245L [134], G98S and L138F [135]). All these variations are missense mutations that do not abrogate protein expression, but constitutively activate SOCE. The diseases that are caused by these mutations are autosomal dominant and include York platelet syndrome, Stormorken syndrome and tubular aggregate myopathy. Symptoms of tubular aggregate myopathy include muscle weakness, myalgia, and cramps; Stormorken and York platelet syndromes present with muscle weakness, miosis, thrombocytopenia, hyposplenism, ichthyosis, dyslexia, and short stature (reviewed in [128,129,136]).

### 1.4. Mitochondrial Calcium Uptake and SOCE Crosstalk

The first link between mitochondrial Ca^2+^ uptake and SOCE was identified well before the molecules that are involved in mediating these processes. It was demonstrated that the pharmacological disruption of the inner mitochondrial membrane (IMM) potential or depletion of sodium (Na^+^) could inhibit SOCE [137]. Indeed, the physiological relevance of this mitochondria-SOCE link was shown in studies comparing nuclear factor of activated T-cells (NFAT) translocation after T-cell activation in the presence and absence of mitochondrial Ca^2+^ uptake diminishers [138]. Mitochondrial Ca^2+^ uptake near store-operated Ca^2+^ channels is believed to prevent Ca^2+^-dependent inactivation by limiting the formation of high Ca^2+^ level domains. Interestingly, one study showed that the distance between mitochondria and PM SOCE channels decreases upon SOCE activation, allowing for a more sustained Ca^2+^ entry due to reduced CDI [139]. In contrast, follow-up work suggested that mitochondrial Ca^2+^ uptake, but not mitochondrial translocation/motility, was required for STIM1 and Orai1-mediated SOCE [140]. Furthermore, respiring mitochondria were independently shown to be required for SOCE activation and were found to regulate slow CDI of SOCE by buffering cytosolic Ca^2+^ levels [141].

Specific links between SOCE and MCU have also been established with MCU knockdown studies showing reduced mitochondrial Ca^2+^ uptake following SOCE [142]. MCU is not the only recently identified mitochondrial protein that has been directly linked to SOCE regulation. The Na^+^/Ca^2+^/lithium (Li^+^) exchanger (NCLX) on the IMM has been shown to be vital for clearance of mitochondrial Ca^2+^ (reviewed in [143]). SOCE is accompanied by a rise in cytosolic Na^+^, which is used by NCLX in order to drive Ca^2+^ efflux from the matrix. The removal of extracellular Na^+^ during SOCE activation inhibits NCLX and SOCE [144]. The activity of NCLX is tightly linked to Orai1 channel inactivation. In the absence of NCLX activity, the loss of Ca^2+^ efflux from the matrix enhances ROS-dependent oxidation of a Cys residue on Orai1, which inhibits SOCE [144]. It is becoming clear that the crosstalk between SOCE and mitochondria involve several different molecules that can both positively and negatively regulate Orai1 channel function [145].

## 2. Mitochondrial Calcium Uniporter (MCU)

Mitochondria are primarily recognized for their role in adenosine triphosphate (ATP) production, a process that requires an electrochemical gradient across the IMM [146]. Mitochondrial Ca^2+^ uptake plays a central role in regulating this energy production and it is also instrumental in regulating apoptosis and shaping cytosolic Ca^2+^ transients [147]. While Ca^2+^ can readily move through the voltage dependent anion channel (VDAC) of the outer mitochondrial membrane, Ca^2+^ uptake into the mitochondrial matrix is more precisely controlled [148]. MCU plays a major role in mediating Ca^2+^ uptake into the matrix, functioning as a multimeric protein complex that forms a Ca^2+^ selective pore through the IMM [12,13]. Under resting conditions, the MCU complex minimally permits the movement of Ca^2+^ into the matrix, despite the highly negative IMM potential (~−180 mV); following a rise in cytosolic Ca^2+^ levels, the MCU channel open probability increases, allowing for Ca^2+^ to move into the matrix, being driven by the IMM potential [146,149,150]. The MCU complex is composed of the channel-forming protein, MCU, which tetramerizes to form a functional pore [151,152,153,154,155], and several additional regulatory protein components. The best studied protein regulators include a family of EF-hand containing proteins, mitochondrial Ca^2+^ uptake 1, 2, and 3 (MICU1, MICU2 and MICU3) [149,156,157]; a small protein that is involved in bridging MICU proteins to the MCU pore, essential MCU regulator (EMRE) [158]; an MCU paralog, MCU dominant negative beta subunit (MCUb) [159]; and a complex stabilizing protein, MCU regulator 1 (MCUR1) [160].

**Figure 3:**
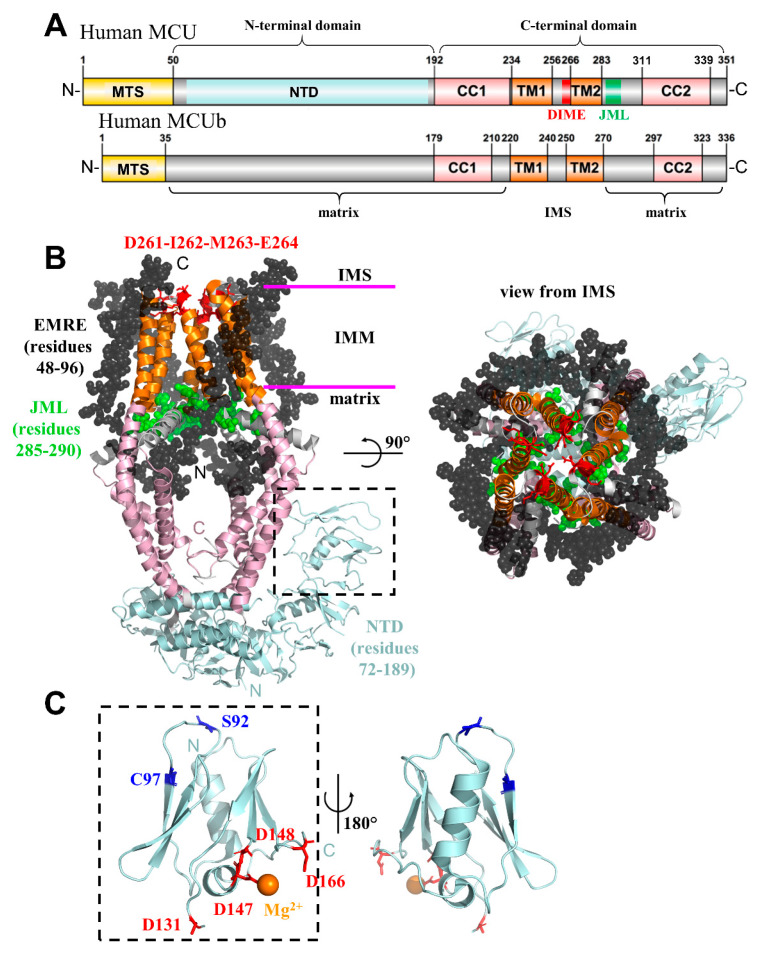
Structural elucidation of the human MCU pore. (**A**) Domain architecture of human mitochondrial Ca^2+^ uniporter (MCU) (Uniprot accession Q8NE86) and MCUb (Uniprot accession Q9NWR8). The conserved coiled-coil and transmembrane regions are shaded salmon and orange, respectively. The residue ranges based on Uniprot annotation are indicated above the respective domain. The topological orientation relative to the inner mitochondrial membrane (IMM) is indicated below the diagrams and the amino (N)-terminal and carboxyl (C)-terminal domains that have been the focus of separate structural studies [162,163,164] are indicated above the diagrams. (**B**) Human MCU tetramer in complex with four essential MCU regulator (EMRE) peptides. The EMRE peptides (black spheres) are oriented with the N-termini in the matrix and the C-termini in the IMS. The EMRE N-termini are situated adjacent to the JML (green spheres), stabilizing the loop conformation. The MCU N- and C-termini are oriented in the matrix. The MCU C-terminal domain (salmon cartoon representation) assembles as a symmetric tetramer, while the N-terminal domain (cyan cartoon representation) exhibits a more linear/crescent tetramer assembly. The Asp-Ile-Met-Glu (DIME) motif (red sticks), important for Ca^2+^ selectivity and permeation, is indicated near the IMS opening of the channel. (**C**) Human MCU-N-terminal domain (MCU-NTD) structure showing the location of various sensory input sites. The glutathionylation C97 and phosphorylation S92 post-translation modification sites (blue sticks) are indicated. The negatively charged Asp residues (red sticks) in close proximity to the Mg^2+^ (orange sphere) binding site are shown. In *B* and *C*, colours of MCU regions are consistent with panel *A*. JML, juxtamembrane loop; IMM, inner mitochondrial membrane; IMS, intermembrane space; NTD, N-terminal domain. The structure figures were made using the 6O58 [154] and 5KUJ [164] pdb coordinate files for the MCU-EMRE complex and MCU-NTD, respectively. MTS, mitochondrial targeting sequence; CC1, -2, coiled-coil-1, -2; TM1, -2, transmembrane-1, -2; N, amino terminus; C, carboxyl terminus.

The MCU pore forming subunit is an ~40 kDa protein (including the mitochondrial targeting sequence; NCBI accession NP_612366.1) composed of two transmembrane domains spanning the IMM, with both the N- and carboxyl (C)-termini localized in the mitochondrial matrix [12,13,161] (Figure 3A). An Asp-Ile-Met-Glu (DIME) motif confers Ca^2+^ selectivity and controls permeability through the channel due to binding of Ca^2+^ to the acidic residues [12,13]. Amino acid substitutions at D261 and E264 lead to a loss of Ca^2+^ uptake, which is likely due to the removal of these negative charges [12,13]. MCU subunits homo-oligomerize to form a functioning Ca^2+^-permeable pore [13]. Initially, a solution nuclear magnetic resonance (NMR)-driven model of *Caenorhabditis elegans* MCU was suggested to exist as a pentamer, with the DIME motifs forming an unstructured loop at the opening of the channel [162]. Several resolved structures since the *C. elegans* NMR model have established metazoan MCU as a tetramer with the DIME motifs lining the pore as part of the helical transmembrane regions [151,152,154] (Figure 3B).

Interestingly, the C-terminal domain of MCU alone can form a channel, because it contains the TM1 and TM2 helices that create the IMM channel pore [151,154,162,163]; however, the N-terminal domain (NTD) of human MCU, which resides in the matrix, has been demonstrated to be an important hub for inputs that regulate the channel function (Figure 3C). For example, the self-association of MCU-NTD promotes channel assembly and activation [164], oxidative modification of C97 within MCU-NTD alters the channel architecture and function [165], and the S92A mutation within MCU-NTD dominant negatively disrupts mitochondrial Ca^2+^ uptake [163]. It is noteworthy that MCU-NTD from lower order organisms assembles as a dimer of dimers directly under the pore [151,152,153,155] and a dimer of crescent-arranged tetramers across two human MCU channels, as revealed by cryo-electron microscopy (cryo-EM) [154].

### 2.1. Essential Mitochondrial Calcium Uniporter Regulator (EMRE)

EMRE is a metazoan-specific ~11 kDa (including the mitochondrial targeting sequence; (NCBI accession NP_201575.3) single-pass membrane protein that spans the IMM [158]. EMRE interacts with MCU at the IMM and with the MICU1/MICU2 heterodimer in the intermembrane space (IMS) [158,166]. Thus, EMRE acts to bridge the MICU1/MICU2 Ca^2+^-sensing properties to MCU channel activity. The presence of an Asp-rich C-terminus is thought to be important in MCU complex regulation [158,166]; however, the specific topology of EMRE has been debated in the past [166,167]. The most recent human MCU complex structure has shown that the EMRE C-terminus is positioned in the IMS [154] (Figure 3B). EMRE is ubiquitously expressed in all tissues, and the knockdown of EMRE leads to the loss of MCU-mediated Ca^2+^ uptake in mammals; subsequent overexpression of MCU is unable to recover the channel function [158,166]. The knockdown of MCU concomitantly reduces EMRE expression levels, indicating a mechanism of co-stabilization [158]. It is important to note that EMRE also plays an integral role in gating MCU by stabilizing the position of an MCU juxta-membrane loop away from the pore exit [154] (Figure 3B).

### 2.2. Mitochondrial Calcium Uptake (MICU) Proteins

MICU1 was the first component of the MCU complex discovered [156]. MICU1 is an ~55 kDa (including the mitochondrial targeting sequence; NCBI accession NP_001350442.1) membrane associated protein, containing two conserved EF-hand Ca^2+^-binding domains [156]. MICU1 is localized to the IMS and indirectly modulates MCU activity via interactions with EMRE [156,158,168]. The initial findings suggested MICU1 functions independently to alter MCU-facilitated Ca^2+^ uptake; however, two additional MICU isoforms, MICU2 and MICU3, have since been discovered to play a role in this Ca^2+^-dependent regulation [157,169]. MICU2 and MICU3 contain the EF-hand Ca^2+^-binding domains that are similarly found in MICU1, despite only sharing 25% sequence identity [157]. MICU1 and MICU2 are ubiquitously expressed in all tissues, while MICU3 appears to be more enriched in the nervous system and skeletal muscle [157,170,171]. MICU1 and MICU2 play synergistic roles in MCU regulation and, together in a complex, function as gatekeepers of the channel [170]. It has been proposed that, at low cytosolic Ca^2+^ levels, MICU1 and MICU2 form a loose dimer, which inactivates MCU [172,173,174,175,176]. Following a rise in cytosolic and IMS Ca^2+^ concentrations, Ca^2+^ binds to the MICU EF-hands and triggers a conformational rearrangement that promotes tighter MICU1/MICU2 interactions and relieves MCU inhibition [172,173,174,175,176]. MICU3 appears to have a specific role in regulating neuronal function and synaptic activity. Specifically, MICU3 is believed to dimerize with MICU1 via disulfide bonds, functioning as a potent enhancer of mitochondrial Ca^2+^ uptake that is controlled by MCU in skeletal muscle and the central nervous system [171].

Several high-resolution crystal structures of MICU1, MICU2, and MICU3 have been elucidated alone or co-complexed and in the presence and absence of Ca^2+^ [173,175,176,177,178]. However, a super-complex cryo-EM structure of human MCU-EMRE-MICU1-MICU2 recently submitted to bioRxiv has suggested that a single MICU1-MICU2 heterodimer regulates the pore exit gate on each channel via Ca^2+^-sensitive MICU1-EMRE contacts [179]. Further, each heterodimer is observed to bridge separate channels via MICU2-MICU2 interactions [179].

### 2.3. Mitochondrial Calcium Uniporter Dominant Negative Beta (MCUb) Subunit

MCUb, an MCU paralog, is an ~39 kDa protein (including the mitochondrial targeting sequence; NCBI accession NP_060388.2) that shares ~50% sequence identity with MCU [159] (Figure 3A). Much like MCU, MCUb has two transmembrane domains, two coiled-coil domains, and matrix-oriented N- and C-termini. Although MCUb contains a similar DIME motif as MCU, MCUb has two key amino acid substitutions (i.e., R251W and E256V) in the pore lining region, which might underlie the inability of MCUb to function as a Ca^2+^-permeable pore [159]. Interestingly, MCU pore subunits have been shown to hetero-oligomerize with MCUb [159]. Mechanistically, MCUb might displace an MCU subunit from the complex to dominant negatively inhibit the channel function [180]. Once incorporated into the MCU pore, MCUb leads to a drastic decrease in mitochondrial Ca^2+^ uptake by diminishing the open probability of the channel and Ca^2+^ permeability [159,180]. Nevertheless, the precise atomic basis by which MCUb inhibits MCU function remains elusive.

### 2.4. Mitochondrial Calcium Uniporter Regulator-1 (MCUR1)

MCUR1 has been identified as a positive regulator of MCU, functioning as a scaffold factor that binds to both MCU and EMRE [149,181]. MCUR1 is an ~40 kDa protein (including the mitochondrial targeting sequence; NCBI accession NCBI: NP_001026883.1), which has two transmembrane domains, two conserved coiled-coil domains, and N- and C-termini residing in the IMS. The interaction between MCU and MCUR1 likely occurs in the mitochondrial matrix, stabilizing the MCU complex [181]. MCUR1 knockdown decreases mitochondrial Ca^2+^ uptake, reduces the IMM potential, and disrupts oxidative phosphorylation [181,182]. Collectively, these data suggest that MCUR1 not only regulates MCU-dependent Ca^2+^ uptake, but also plays a key role in controlling cellular bioenergetics.

### 2.5. MCU and Disease

Mitochondrial Ca^2+^ signaling has been implicated in a variety of pathophysiological disorders. Aberrant mitochondrial Ca^2+^ uptake not only affects processes reliant on maintaining tight cytosolic Ca^2+^ concentrations, but also impacts oxidative metabolism and can trigger cell death pathways [146,147,183]. Under cell stress conditions, mitochondrial Ca^2+^ overload and/or oxidative stress results in an opening of the mitochondrial permeability transition pore (mPTP) [183,184]. High conductance mPTP allows the free movement of ions and small molecules less than ~1.5 kDa, dissipating the IMM potential and aberrating ATP production [185,186]. Ultimately, this type of mPTP opening leads to the swelling and rupture of mitochondria and release of larger pro-apoptotic factors [183,184]. Thus, dysregulated mitochondrial Ca^2+^ uptake has dire implications for cell survival and has been associated with various diseases, including cancer, neurodegenerative pathologies, diabetes, and learning and muscular disorders, among others [187,188,189,190,191].

Patients have been identified with heritable *MICU1* mutations (i.e., NM_006077.3:c.1078-1G>C and NM_006077.3:c.741+1G>A in splice donor and acceptor sites, respectively), which result in intronic insertions causing frameshifts, nonsense mediated mRNA decay, and loss of MICU1 protein [187,192,193]. Patient cohorts harboring *MICU1* exon 1 deletions (2,776 nucleotides) [194] and *MICU1* nonsense mutations (i.e., NM_006077.3:c.553C>T:p.Q185* [195]) have been identified, also abolishing the MICU1 protein. Similarly, a heritable *MICU2* nonsense mutation (i.e., NM_152726:c.42G>A:p.W14*) has been discovered, which eliminates full-length MICU2 protein [196]. All of these heritable mutations lead to loss of function MICU disorders, characterized by muscle weakness, fatigue, lethargy, developmental delay, and learning disabilities [193,194,195,196]. Patient fibroblasts with MICU1 protein abrogation have conflictingly demonstrated both increased [193] and decreased [194] rates of mitochondrial Ca^2+^ uptake. Further, MICU2 protein abrogation also suppressed mitochondrial Ca^2+^ uptake rates [196]. Nevertheless, all of the patient studies have shown enhanced resting mitochondrial Ca^2+^ levels due to either MICU1 or MICU2 protein downregulation [193,194,196], which is likely caused by the absence of MCU gatekeeping.

Enhanced mitochondrial Ca^2+^ uptake can suppress cytosolic Ca^2+^ signals in fibroblasts from MICU1-deficient patients [193], which is consistent with past studies showing mitochondria can suppress cytosolic Ca^2+^ signals [197,198,199,200]. Further, this enhanced mitochondrial Ca^2+^ uptake may be related to work showing deletion of MICU1 in mouse hepatocytes causes sensitization to Ca^2+^-overload-induced mPTP opening [201]. The identification of heritable mutations in MCU complex components that lead to disease underscore the importance of not only the MCU channel, but also the diverse regulatory controls of MCU function.

## 3. Leucine Zipper EF-Hand Containing Transmembrane Protein-1 (LETM1)

LETM1 is an essential IMM protein linked to mitochondrial ion transport, regulation of cell cycle, mitochondrial oxidative stress and bioenergetic function [202]. Interestingly, LETM1 has been shown to play a role in mitochondrial Ca^2+^ and K^+^ ion homeostasis, regulating key facets of mitochondrial physiology, such as osmotic balance and ATP production [14,203,204,205,206,207,208]. While the molecular mechanisms by which LETM1 functions remain incompletely understood, it is clear that LETM1 is pivotal in mitochondrial function and cellular health. The deletion of the LETM1 homologue in yeast, MDM38, results in mitochondrial swelling, loss of cristae, and disruption of cellular respiration [205]. Homozygous LETM1 deletion is embryonically lethal within ~6 days in mice [204]. Clinically, LETM1 haploinsufficiency in humans is thought to be responsible for seizures in patients with Wolf–Hirschhorn syndrome (WHS) [209,210] (see below). Consistent with these findings, two independent studies identified *LETM1* as one of ~2000 essential genes in human cell cultures [211,212].

In the context of mitochondrial Ca^2+^ ion transport, while MCU facilitates low affinity/high throughput Ca^2+^ uptake triggered at ~µM cytosolic Ca^2+^ levels (see above), LETM1 has been shown to transport Ca^2+^ at ~nM cytosolic Ca^2+^ levels, suggesting that LETM1 provides cells with a high affinity/low capacity pathway necessary for precise mitochondrial Ca^2+^ homeostasis [208,213,214,215]. Indeed, liposome reconstitution assays and cell culture work have shown that LETM1 directly facilitates selective bidirectional Ca^2+^/H^+^ exchange, independent of MCU [203,206,208]. Congruently, LETM1 knockdown decreases mitochondrial Ca^2+^ uptake, decreases ATP production, and increases ROS production [203,216].

**Figure 4:**
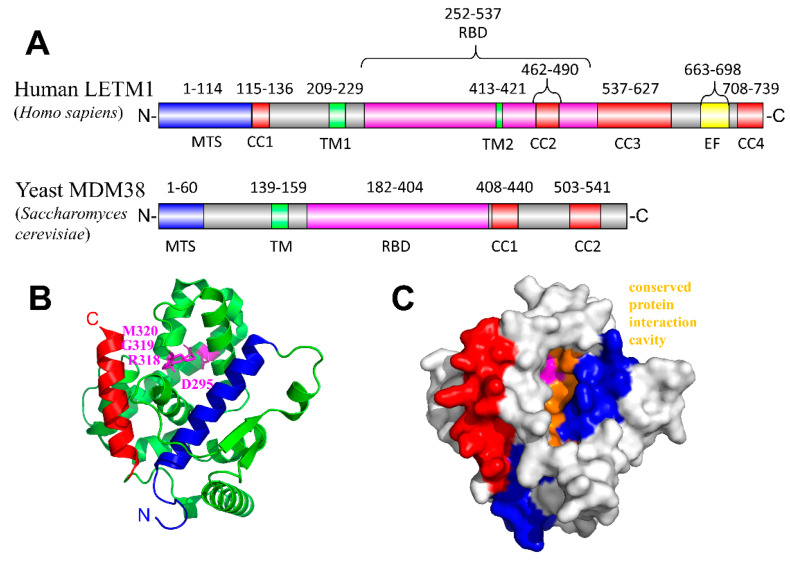
Structural insights into potential LETM1 interaction interfaces. (**A**) Domain architecture of human LETM1 (Uniprot accession O95202) and the yeast LETM1 homologue (MDM38; Uniprot accession Q08179). The predicted/putative location of four coiled-coil domains (red), two transmembranes helices (green), EF-hand Ca^2+^-binding motif (yellow), mitochondrial targeting sequence (blue), ribosome binding domain (magenta) and 14-3-3 like domain (cyan) are indicated with residue ranges shown above the respective segment based on the Uniprot annotation. (**B**) Yeast MDM38 ribosome binding domain crystal structure exhibiting a 14-3-3 like fold. The amino (N)-terminal and carboxyl (**C**) terminal helices are coloured blue and red, respectively. The N-terminal helix has been previously implicated in mediating protein-protein interactions of the structurally homologous human 14-3-3 epsilon protein [219]. The conserved residue positions necessary for functional human LETM1 assembly and growth complementation of yeast deficient in MDM38 [220] are coloured magenta (i.e., D295, R318, G319, M320 in yeast MDM38; shown as sticks). (**C**) Surface representation of the yeast MDM38 ribosome binding domain showing a putative substrate/protein interaction cavity (orange), which may interact with phosphorylated proteins akin to human 14-3-3 structural homologues [219]. CC1 -2, -3, 4, coiled-coil-1, -2, -3; TM1, -2, transmembrane-1, -2; EF, EF-hand; RBD, ribosome binding domain; MTS, mitochondrial targeting sequence. The structure figures were made using the 3SKQ [219] pdb coordinate file for yeast MDM38 ribosome binding domain.

Human LETM1 is an ~83 kDa transmembrane protein (including the mitochondrial targeting sequence; NCBI accession NP_036450.1) that localizes in the IMM [206,207] (Figure 4A). LETM1 is thought to topologically orient with the N-terminus facing the IMS. This IMS region includes a putative coiled-coil domain and a notable PTEN-induced kinase 1 (PINK1) mediated threonine phosphorylation site [217]. LETM1 contains a single sequence-identifiable transmembrane domain and a large matrix-oriented C-terminal region, which includes a putative ribosome binding domain, coiled-coil domains, and a Ca^2+^-binding EF-hand motif [15]. This topology for LETM1 is consistent with restricted proteinase K digestion studies [204,206,207]. However, a recent study using probes targeting tyrosine residues has proposed a new topology for LETM1, where two transmembrane domains exist and both the N- and C-termini are located in the matrix [218] (Figure 4A).

LETM1 self-oligomerizes and directly facilitates selective Ca^2+^/H^+^ exchange within liposomes without any accessory proteins [204,206,208]. A low-resolution EM study suggests human LETM1 forms a hexamer, and pH may modulate the conformation [206]. Interestingly, the crystal structure of the C-terminal domain of the yeast homologue MDM38 has been solved and exhibits a 14-3-3 like protein interaction domain, which might indicate potential sites for inter- or intra-protomer binding [219,220] (Figure 4B,C). It is tempting to speculate that these interfaces could be targeted by drugs and small molecules aimed at modulating LETM1 assembly and function.

### LETM1 and Disease

Located on the chromosome band 4p16.3, *LETM1* is included in one of two critical regions lost in almost all patients exhibiting the full phenotype of WHS, which causes delayed growth and development, microcephaly, distinctive facial abnormalities, and seizures [204,209]. Deficiency in mitochondrial Ca^2+^ transport, decreased ATP levels, and particularly increased ROS may be key determinants of the neurodegeneration linked to seizures in this disease [210]. Given that loss of LETM1 suppresses ATP production, enhances ROS, and inhibits cell proliferation [221], it is no surprise the overexpression of LETM1 has been found in many cancers, including ovarian, rectal, stomach, esophagus, breast, colon, and lung non-small cell carcinoma [222,223,224].

## 4. Generating New Therapeutics and Diagnostics from Protein Structures

Over the past ~15 years, a number of protein structures involved in regulating cytosolic and stored Ca^2+^ have been elucidated. This surge of structural information has not only been fueled by high throughput proteomic and genomic approaches that have solved decades old mysteries regarding the molecular identities of proteins that are involved in mediating ubiquitous cellular processes, such as SOCE [30,31] and mitochondrial Ca^2+^ uptake [12,13], but also by improving technologies aimed at revealing protein structures, such as cryo-EM, solution NMR, and X-ray crystallography. For example, several high-resolution structures of the molecular machinery involved in mediating SOCE have been elucidated, including the STIM Ca^2+^-sensing domains by solution NMR [75,113], the cytosolic coiled-coil domains by solution NMR [107], and X-ray crystallography [106,108] (Figure 1), as well as the Orai1 Ca^2+^ channel itself by cryo-EM and X-ray crystallography [120,225,226]. Cryo-EM has driven medium/high-resolution structures of MCU from lower order and metazoan organisms, including humans [151,152,153,154,155] (Figure 3). Importantly, X-ray crystallography has also contributed to understanding the underlying mechanisms of MCU regulation with elucidations of the important gatekeeping proteins [173,175,176,177,178], environment-specific conformations [163,164,227], and scaffolds [154,228]. The first elucidations of IP_3_Rs by crystallography dates back almost two decades with structures of the N-terminal domain [229,230]. The analogous N-terminal region in RyR has also been studied by both crystallography and NMR [231,232]. In fact, crystal structures of the full N-terminal domains of both IP_3_Rs and RyRs revealed a remarkable structural and functional conservation between these receptor cousins at the apical positions of the receptors [233,234]. These tetrameric channels have also been excellent candidates for cryo-EM, due to their inherently large size [235,236,237]. Other Ca^2+^ toolkit components, such as Na^+^/Ca^2+^ exchangers (NCX) [238], S/ER Ca^2+^ ATPase (SERCA) pumps [239,240], and VGICs, have also been structurally resolved [241,242]. This list of structures only scratches the surface of the available Ca^2+^ toolkit structural data, yet reinforces a major question at the nexus of the structural biology, cell biology, and clinical research fields. How can we use this wealth of protein structural information to design new diagnostic tools and drugs for research and treatment of disease?

Indeed, while obtaining structural information has been invaluable for informing the broad scientific community on the molecular and structural basis for the function of hundreds or thousands of Ca^2+^ signaling proteins, there is a disconnect between applying this protein structural knowledge and rationally developing research tools or drugs. Perhaps this disconnect exists due to the relatively static insights provided by many of the resolved structures. Most (if not all) protein function involves dynamic changes in conformation and stability. Rationalizing effective drugs without a detailed understanding of the conformational ensembles present in the inactive, transition, and active states of proteins is practically challenging.

### 4.1. Store-Operated and Mitochondrial Ca^2+^ Entry Proteins as Drug Targets

The SOCE and mitochondrial Ca^2+^ uptake protein machinery are appealing drug targets for several reasons. First, mutations causing disease have been identified in the proteins mediating these processes (see above). Second, complexes of multiple different proteins regulate SOCE and mitochondrial Ca^2+^ uptake; hence, the modulation of function, in some cases, could be achieved by targeting native protein interaction partner components. Third, drugs targeting ion channels are demonstrated to be effective at treating a wide range of human diseases and acute conditions [243,244,245]. Fourth, clinically approved immunosuppressive drugs already exist that block signaling processes that are directly mediated by SOCE (e.g., cyclosporin [246,247]). Finally, the acute administration of MCU inhibitors is known to protect cells from hypoxia/ischemia-induced injury [248,249,250,251]. The specific structures available for the SOCE and mitochondrial Ca^2+^ uptake molecular machinery provide a unique opportunity for the development of new diagnostics and/or therapeutics that can biasedly target different aspects of these pathways.

Small molecule screens targeting MCU function have already been performed. An initial study employed *Saccharomyces cerevisiae* as an MCU-null organism to screen for small molecule modulators of MCU function. Human MCU, human EMRE, and a mitochondrial-targeted Ca^2+^ indicator (i.e., aequorin) were expressed in these yeasts to establish a reporter system for the screening of ~600 clinically approved drugs [252]. In a second approach, HEK293A cells expressing mitochondrial aequorin were used to screen a 120,000 compound library, successfully identifying an MCU inhibitor [253]. In the most recently published screen, HeLa cells expressing mitochondrially-targeted aequorin were used to identify two MCU inhibitors from a library of 44,000 compounds [254]. All of these in cellulo screens used genetically encoded aequorin, reliably targeted to the matrix, for luminescence readout of changes in Ca^2+^.

Since the identification of STIM1 and Orai1, published drug screens targeting SOCE have largely involved the over-expression of STIM1 and Orai1 in mammalian cells or the use of cells with endogenous levels of STIM1 and Orai1, in combination with indicator dyes or proteins monitoring cytosolic Ca^2+^ or NFAT translocation to the nucleus [255,256,257,258]. One in vitro study used recombinant functional domains to screen for binding to immobilized small molecules by immunofluorescence [259]. In silico tools have also been used, but only to identify drug candidates through small molecule homology to known SOCE inhibitors [260,261].

Thus, while drug screens targeting the protein machinery that mediates both store-operated and mitochondrial Ca^2+^ uptake have been performed, these published approaches have not used the available protein structure information as initiation points to drug discovery.

### 4.2. Initiating Protein Structure-based Drug Discovery

A starting point for drug development could be initiated by a simple high-throughput small molecule in silico screen of millions of compounds. This computational screen would use available protein structures and knowledge of the vital interfaces modulating activity. The structures of the small molecules/drugs would also need to be known. Such small molecule databases and computational docking programs exist and are freely available (e.g., see ZINC15 [262] and AUTODOCK [263]). The major bottleneck for this initial step is computing the resources that are required to dock millions of compounds in a relatively short time. Nevertheless, once top leads are identified, subsequent wet laboratory experiments are required in order to validate the interaction and physiological effect(s). Downstream structure-activity-relationship studies could be subsequently used to better understand the small molecule chemical groups that are required for binding and eliciting a cellular effect and improving the compound [264].

Of course, there are limitations to these large-scale computational screens. For example, the protein structures are generally treated as rigid bodies, unless using higher level molecular dynamics simulations. Water and/or lipid molecules are typically not modeled in these fast docking schemes, even though these molecules play integral roles in biological interactions. Most critically, default scoring schemes may not be optimal for a system under study, since not all of the interactions equally rely on specific stabilization forces (e.g., H-bonding, hydrophobic, charge, shape complementarity).

High resolution structural information can also be used to develop tools for high-throughput drug discovery. For example, fluorescence-resonance energy transfer (FRET)-based biosensors can be rationalized based on proximity information that is revealed by isolated and co-complexed structures. With respect to Ca^2+^ signaling, these types of FRET-based sensors have been engineered and used in screens for the identification of SERCA and RyR modulators. A green fluorescent protein (GFP) and red fluorescent protein (RFP) FRET pair was fused into the cytosolic headpiece of SERCA, which undergoes large scale structural changes during pump activity. This intramolecular FRET sensor was successfully used to identify the small molecule modulators of SERCA function [265,266]. Since the sensor is genetically encoded, the cell lines stably expressing this sensor could be used in the screening. An analogous approach was used by the same group to identify modulators of RyR function. However, the RyR screen relied on FRET between FK binding protein 12.6 (FKBP12) and calmodulin, which co-complex with RyR and show different FRET signals that are based on RyR conformation [267,268]. In these RyR experiments, Alexa Fluors were chemically linked to FKBP12 and calmodulin in vitro, and vesicles containing RyRs were prepared from muscle tissues. Two aspects of STIM/Orai and MCU signaling make this approach to identifying drugs particularly appealing: (i) these proteins function in large complexes that are amenable to intermolecular FRET monitoring and (ii) STIMs are known to undergo large conformational changes, making them amenable to intramolecular FRET monitoring.

### 4.3. Concluding Remarks

Not discussed herein are the countless additional experimental (involving molecules, cells, animals, and humans), logistical, and administrative hurdles that are required to move any small molecule lead into clinical use. Nevertheless, we provide a simple strategy for applying high-resolution structural knowledge of proteins as a starting point to drug development. Excitingly, several key protein structures are available that inform on the mechanisms regulating the SOCE-mitochondrial Ca^2+^ signaling axis, providing several possible initiating points to protein structure-based drug design. The elucidation of Ca^2+^ signaling protein structures is vital for understanding the fundamental mechanisms underlying cellular processes that are integral to life and death. The structural information can also provide an entry point for the development of tools and therapeutics targeting these pathways.

## Figures and Tables

**Table 1:** Various signaling mechanisms leading to sarco/endoplasmic reticulum (S/ER) Ca^2+^ store release.

Surface Receptor or Channel	Extracellular Signal	Immediate Effect of Receptor Activation	Downstream S/ER Ca^2+^ Release Channel	References
GPCR (e.g., PAR1)	Protein and small molecule ligands (e.g., thrombin)	PLCβ activation	IP_3_R	[38,39,40,41,42]
^a^ Polymodal GPCR (e.g., M_3_R and PTHR1)	Protein and small molecule ligands (e.g., carbachol/PTH)	PLCβ and adenylyl cyclase activation	IP_3_R	[43,44,45]
RTK (e.g., IGF-1R)	Protein ligands (e.g., IGF-1)	PLCγ activation	IP_3_R	[46,47,48]
^b^ BCR	IgM-binding antigens	PLCγ activation	IP_3_R	[49,50,51,52]
^b^ TCR	Antigen presenting cell (MHC)	PLCγ activation	IP_3_R	[16,17,53,54]
Fc	Antigen-antibody complex	PLCγ activation	IP_3_R	[16,17,55,56]
VGIC (e.g., L-type Ca^2+^ channel)	Membrane depolarization	Ca^2+^ influx from the extracellular space	RyR	[57,58,59,60,61]

**^a^** Simultaneous distinct GPCR stimuli can lead to the production of IP_3_ (via PLC) and cAMP (via adenylyl cyclase). cAMP sensitizes a subset of high affinity IP_3_Rs in a discrete S/ER Ca^2+^ store to IP_3_, which would otherwise remain inactive in the absence of cAMP. **^b^** TCR and BCR activation can also lead to the generation cyclic adenosine diphosphate ribose (cADPr), resulting in S/ER Ca^2+^ release via RyR activation [16,17,62,63,64]. PTH, parathyroid hormone; PTHR1, PTH receptor type-1; cAMP, 3′,5′-cyclic adenosine monophosphate; M3R, muscarinic type-3 acetylcholine receptor.

## References

[1] Kirichok Y., Krapivinsky G., Clapham D.E. (2004). The mitochondrial calcium uniporter is a highly selective ion channel. Nature.

[2] Pinton P., Giorgi C., Siviero R., Zecchini E., Rizzuto R. (2008). Calcium and apoptosis: ER-mitochondria Ca2+ transfer in the control of apoptosis. Oncogene.

[3] Rimessi A., Giorgi C., Pinton P., Rizzuto R. (2008). The versatility of mitochondrial calcium signals: From stimulation of cell metabolism to induction of cell death. Biochim. Biophys. Acta.

[4] Deluca H.F., Engstrom G.W. (1961). Calcium uptake by rat kidney mitochondria. Proc. Natl. Acad. Sci. USA.

[5] Amberger A., Weiss H., Haller T., Kock G., Hermann M., Widschwendter M., Margreiter R. (2001). A subpopulation of mitochondria prevents cytosolic calcium overload in endothelial cells after cold ischemia/reperfusion. Transplantation.

[6] Ly L.D., Ly D.D., Nguyen N.T., Kim J.H., Yoo H., Chung J., Lee M.S., Cha S.K., Park K.S. (2020). Mitochondrial Ca(2+) Uptake Relieves Palmitate-Induced Cytosolic Ca(2+) Overload in MIN6 Cells. Mol. Cells.

[7] Yi M., Weaver D., Hajnoczky G. (2004). Control of mitochondrial motility and distribution by the calcium signal: A homeostatic circuit. J. Cell Biol..

[8] Peng T.I., Jou M.J. (2010). Oxidative stress caused by mitochondrial calcium overload. Ann. N. Y. Acad. Sci..

[9] Patron M., Raffaello A., Granatiero V., Tosatto A., Merli G., De Stefani D., Wright L., Pallafacchina G., Terrin A., Mammucari C. (2013). The mitochondrial calcium uniporter (MCU): Molecular identity and physiological roles. J. Biol. Chem..

[10] Cardenas C., Miller R.A., Smith I., Bui T., Molgo J., Muller M., Vais H., Cheung K.H., Yang J., Parker I. (2010). Essential regulation of cell bioenergetics by constitutive InsP3 receptor Ca2+ transfer to mitochondria. Cell.

[11] Jouaville L.S., Pinton P., Bastianutto C., Rutter G.A., Rizzuto R. (1999). Regulation of mitochondrial ATP synthesis by calcium: Evidence for a long-term metabolic priming. Proc. Natl. Acad. Sci. USA.

[12] Baughman J.M., Perocchi F., Girgis H.S., Plovanich M., Belcher-Timme C.A., Sancak Y., Bao X.R., Strittmatter L., Goldberger O., Bogorad R.L. (2011). Integrative genomics identifies MCU as an essential component of the mitochondrial calcium uniporter. Nature.

[13] De Stefani D., Raffaello A., Teardo E., Szabo I., Rizzuto R. (2011). A forty-kilodalton protein of the inner membrane is the mitochondrial calcium uniporter. Nature.

[14] Jiang D., Zhao L., Clapham D.E. (2009). Genome-wide RNAi screen identifies Letm1 as a mitochondrial Ca2+/H+ antiporter. Science.

[15] Lin Q.T., Stathopulos P.B. (2019). Molecular Mechanisms of Leucine Zipper EF-Hand Containing Transmembrane Protein-1 Function in Health and Disease. Int. J. Mol. Sci..

[16] Feske S. (2007). Calcium signalling in lymphocyte activation and disease. Nat. Rev. Immunol..

[17] Feske S., Skolnik E.Y., Prakriya M. (2012). Ion channels and transporters in lymphocyte function and immunity. Nat. Rev. Immunol..

[18] Luik R.M., Wang B., Prakriya M., Wu M.M., Lewis R.S. (2008). Oligomerization of STIM1 couples ER calcium depletion to CRAC channel activation. Nature.

[19] Montero M., Barrero M.J., Torrecilla F., Lobaton C.D., Moreno A., Alvarez J. (2001). Stimulation by thimerosal of histamine-induced Ca(2+) release in intact HeLa cells seen with aequorin targeted to the endoplasmic reticulum. Cell Calcium.

[20] Suzuki J., Kanemaru K., Ishii K., Ohkura M., Okubo Y., Iino M. (2014). Imaging intraorganellar Ca2+ at subcellular resolution using CEPIA. Nat. Commun..

[21] Yu R., Hinkle P.M. (2000). Rapid turnover of calcium in the endoplasmic reticulum during signaling. Studies with cameleon calcium indicators. J. Biol. Chem..

[22] Berridge M.J., Lipp P., Bootman M.D. (2000). The versatility and universality of calcium signalling. Nat. Rev. Mol. Cell Biol..

[23] Stathopulos P.B., Seo M.D., Enomoto M., Amador F.J., Ishiyama N., Ikura M. (2012). Themes and variations in ER/SR calcium release channels: Structure and function. Physiology (Bethesda).

[24] Amador F.J., Stathopulos P.B., Enomoto M., Ikura M. (2013). Ryanodine receptor calcium release channels: Lessons from structure-function studies. FEBS J..

[25] Fedorenko O.A., Popugaeva E., Enomoto M., Stathopulos P.B., Ikura M., Bezprozvanny I. (2014). Intracellular calcium channels: Inositol-1,4,5-trisphosphate receptors. Eur. J. Pharmacol..

[26] Stathopulos P.B., Ikura M. (2017). Store operated calcium entry: From concept to structural mechanisms. Cell Calcium.

[27] Novello M.J., Zhu J., Feng Q., Ikura M., Stathopulos P.B. (2018). Structural elements of stromal interaction molecule function. Cell Calcium.

[28] Putney J.W. (1986). A model for receptor-regulated calcium entry. Cell Calcium.

[29] Smyth J.T., Dehaven W.I., Jones B.F., Mercer J.C., Trebak M., Vazquez G., Putney J.W. (2006). Emerging perspectives in store-operated Ca2+ entry: Roles of Orai, Stim and TRP. Biochim. Biophys. Acta.

[30] Liou J., Kim M.L., Heo W.D., Jones J.T., Myers J.W., Ferrell J.E., Meyer T. (2005). STIM is a Ca2+ sensor essential for Ca2+-store-depletion-triggered Ca2+ influx. Curr. Biol..

[31] Roos J., DiGregorio P.J., Yeromin A.V., Ohlsen K., Lioudyno M., Zhang S., Safrina O., Kozak J.A., Wagner S.L., Cahalan M.D. (2005). STIM1, an essential and conserved component of store-operated Ca2+ channel function. J. Cell Biol..

[32] Feske S., Gwack Y., Prakriya M., Srikanth S., Puppel S.H., Tanasa B., Hogan P.G., Lewis R.S., Daly M., Rao A. (2006). A mutation in Orai1 causes immune deficiency by abrogating CRAC channel function. Nature.

[33] Prakriya M., Feske S., Gwack Y., Srikanth S., Rao A., Hogan P.G. (2006). Orai1 is an essential pore subunit of the CRAC channel. Nature.

[34] Vig M., Beck A., Billingsley J.M., Lis A., Parvez S., Peinelt C., Koomoa D.L., Soboloff J., Gill D.L., Fleig A. (2006). CRACM1 multimers form the ion-selective pore of the CRAC channel. Curr. Biol..

[35] Vig M., Peinelt C., Beck A., Koomoa D.L., Rabah D., Koblan-Huberson M., Kraft S., Turner H., Fleig A., Penner R. (2006). CRACM1 is a plasma membrane protein essential for store-operated Ca2+ entry. Science.

[36] Yeromin A.V., Zhang S.L., Jiang W., Yu Y., Safrina O., Cahalan M.D. (2006). Molecular identification of the CRAC channel by altered ion selectivity in a mutant of Orai. Nature.

[37] Hohendanner F., McCulloch A.D., Blatter L.A., Michailova A.P. (2014). Calcium and IP3 dynamics in cardiac myocytes: Experimental and computational perspectives and approaches. Front. Pharmacol..

[38] Hilger D., Masureel M., Kobilka B.K. (2018). Structure and dynamics of GPCR signaling complexes. Nat. Struct. Mol. Biol..

[39] Kadamur G., Ross E.M. (2013). Mammalian phospholipase C. Annu. Rev. Physiol..

[40] Smrcka A.V., Fisher I. (2019). G-protein betagamma subunits as multi-functional scaffolds and transducers in G-protein-coupled receptor signaling. Cell Mol. Life Sci..

[41] Nieman M.T. (2016). Protease-activated receptors in hemostasis. Blood.

[42] Scarlata S. (2019). The role of phospholipase Cbeta on the plasma membrane and in the cytosol: How modular domains enable novel functions. Adv. Biol. Regul..

[43] Konieczny V., Tovey S.C., Mataragka S., Prole D.L., Taylor C.W. (2017). Cyclic AMP Recruits a Discrete Intracellular Ca(2+) Store by Unmasking Hypersensitive IP3 Receptors. Cell. Rep..

[44] Meena A., Tovey S.C., Taylor C.W. (2015). Sustained signalling by PTH modulates IP3 accumulation and IP3 receptors through cyclic AMP junctions. J. Cell Sci..

[45] Tovey S.C., Taylor C.W. (2013). Cyclic AMP directs inositol (1,4,5)-trisphosphate-evoked Ca2+ signalling to different intracellular Ca2+ stores. J. Cell Sci..

[46] Du Z., Lovly C.M. (2018). Mechanisms of receptor tyrosine kinase activation in cancer. Mol. Cancer.

[47] Lemmon M.A., Schlessinger J. (2010). Cell signaling by receptor tyrosine kinases. Cell.

[48] Trenker R., Jura N. (2020). Receptor tyrosine kinase activation: From the ligand perspective. Curr. Opin. Cell Biol..

[49] Feng Y., Wang Y., Zhang S., Haneef K., Liu W. (2020). Structural and immunogenomic insights into B-cell receptor activation. J. Genet. Genom..

[50] Treanor B. (2012). B-cell receptor: From resting state to activate. Immunology.

[51] Kim Y.J., Sekiya F., Poulin B., Bae Y.S., Rhee S.G. (2004). Mechanism of B-cell receptor-induced phosphorylation and activation of phospholipase C-gamma2. Mol. Cell Biol..

[52] Mahtani T., Treanor B. (2019). Beyond the CRAC: Diversification of ion signaling in B cells. Immunol. Rev..

[53] Schamel W.W., Alarcon B., Minguet S. (2019). The TCR is an allosterically regulated macromolecular machinery changing its conformation while working. Immunol. Rev..

[54] Xu X., Li H., Xu C. (2020). Structural understanding of T cell receptor triggering. Cell Mol. Immunol..

[55] Olivera A., Beaven M.A., Metcalfe D.D. (2018). Mast cells signal their importance in health and disease. J. Allergy Clin. Immunol..

[56] Bournazos S., Ravetch J.V. (2015). Fcgamma receptor pathways during active and passive immunization. Immunol. Rev..

[57] Rougier J.S., Abriel H. (2016). Cardiac voltage-gated calcium channel macromolecular complexes. Biochim. Biophys. Acta.

[58] Wu J., Yan N., Yan Z. (2017). Structure-Function Relationship of the Voltage-Gated Calcium Channel Cav1.1 Complex. Adv. Exp. Med. Biol..

[59] Pallien T., Klussmann E. (2020). New aspects in cardiac L-type Ca2+ channel regulation. Biochem. Soc. Trans..

[60] Eisner D.A., Caldwell J.L., Kistamas K., Trafford A.W. (2017). Calcium and Excitation-Contraction Coupling in the Heart. Circ. Res..

[61] Thul R. (2014). Translating intracellular calcium signaling into models. Cold Spring Harb. Protoc..

[62] Guse A.H., da Silva C.P., Berg I., Skapenko A.L., Weber K., Heyer P., Hohenegger M., Ashamu G.A., Schulze-Koops H., Potter B.V. (1999). Regulation of calcium signalling in T lymphocytes by the second messenger cyclic ADP-ribose. Nature.

[63] Kiselyov K., Shin D.M., Shcheynikov N., Kurosaki T., Muallem S. (2001). Regulation of Ca2+-release-activated Ca2+ current (Icrac) by ryanodine receptors in inositol 1,4,5-trisphosphate-receptor-deficient DT40 cells. Biochem. J..

[64] Schwarzmann N., Kunerth S., Weber K., Mayr G.W., Guse A.H. (2002). Knock-down of the type 3 ryanodine receptor impairs sustained Ca2+ signaling via the T cell receptor/CD3 complex. J. Biol. Chem..

[65] Smrcka A.V. (2015). Regulation of phosphatidylinositol-specific phospholipase C at the nuclear envelope in cardiac myocytes. J. Cardiovasc. Pharmacol..

[66] Siltari A., Korpela R., Vapaatalo H. (2016). Bradykinin -induced vasodilatation: Role of age, ACE1-inhibitory peptide, mas- and bradykinin receptors. Peptides.

[67] Thangam E.B., Jemima E.A., Singh H., Baig M.S., Khan M., Mathias C.B., Church M.K., Saluja R. (2018). The Role of Histamine and Histamine Receptors in Mast Cell-Mediated Allergy and Inflammation: The Hunt for New Therapeutic Targets. Front. Immunol..

[68] Galvin C.D., Hardiman O., Nolan C.M. (2003). IGF-1 receptor mediates differentiation of primary cultures of mouse skeletal myoblasts. Mol. Cell. Endocrinol..

[69] Molhoek K.R., Shada A.L., Smolkin M., Chowbina S., Papin J., Brautigan D.L., Slingluff C.L. (2011). Comprehensive analysis of receptor tyrosine kinase activation in human melanomas reveals autocrine signaling through IGF-1R. Melanoma Res..

[70] Goodnow C.C., Sprent J., Fazekas de St Groth B., Vinuesa C.G. (2005). Cellular and genetic mechanisms of self tolerance and autoimmunity. Nature.

[71] Cai X. (2007). Molecular evolution and functional divergence of the Ca(2+) sensor protein in store-operated Ca(2+) entry: Stromal interaction molecule. PLoS ONE.

[72] Enomoto M., Nishikawa T., Back S.I., Ishiyama N., Zheng L., Stathopulos P.B., Ikura M. (2020). Coordination of a Single Calcium Ion in the EF-hand Maintains the Off State of the Stromal Interaction Molecule Luminal Domain. J. Mol. Biol..

[73] Stathopulos P.B., Li G.Y., Plevin M.J., Ames J.B., Ikura M. (2006). Stored Ca2+ depletion-induced oligomerization of stromal interaction molecule 1 (STIM1) via the EF-SAM region: An initiation mechanism for capacitive Ca2+ entry. J. Biol. Chem..

[74] Muik M., Fahrner M., Schindl R., Stathopulos P., Frischauf I., Derler I., Plenk P., Lackner B., Groschner K., Ikura M. (2011). STIM1 couples to ORAI1 via an intramolecular transition into an extended conformation. EMBO J..

[75] Stathopulos P.B., Zheng L., Li G.Y., Plevin M.J., Ikura M. (2008). Structural and mechanistic insights into STIM1-mediated initiation of store-operated calcium entry. Cell.

[76] Zhou Y., Srinivasan P., Razavi S., Seymour S., Meraner P., Gudlur A., Stathopulos P.B., Ikura M., Rao A., Hogan P.G. (2013). Initial activation of STIM1, the regulator of store-operated calcium entry. Nat. Struct. Mol. Biol..

[77] Baba Y., Hayashi K., Fujii Y., Mizushima A., Watarai H., Wakamori M., Numaga T., Mori Y., Iino M., Hikida M. (2006). Coupling of STIM1 to store-operated Ca2+ entry through its constitutive and inducible movement in the endoplasmic reticulum. Proc. Natl. Acad. Sci. USA.

[78] Liou J., Fivaz M., Inoue T., Meyer T. (2007). Live-cell imaging reveals sequential oligomerization and local plasma membrane targeting of stromal interaction molecule 1 after Ca2+ store depletion. Proc. Natl. Acad. Sci. USA.

[79] Luik R.M., Wu M.M., Buchanan J., Lewis R.S. (2006). The elementary unit of store-operated Ca2+ entry: Local activation of CRAC channels by STIM1 at ER-plasma membrane junctions. J. Cell Biol..

[80] Wu M.M., Buchanan J., Luik R.M., Lewis R.S. (2006). Ca2+ store depletion causes STIM1 to accumulate in ER regions closely associated with the plasma membrane. J. Cell Biol..

[81] Muik M., Frischauf I., Derler I., Fahrner M., Bergsmann J., Eder P., Schindl R., Hesch C., Polzinger B., Fritsch R. (2008). Dynamic coupling of the putative coiled-coil domain of ORAI1 with STIM1 mediates ORAI1 channel activation. J. Biol. Chem..

[82] Park C.Y., Hoover P.J., Mullins F.M., Bachhawat P., Covington E.D., Raunser S., Walz T., Garcia K.C., Dolmetsch R.E., Lewis R.S. (2009). STIM1 clusters and activates CRAC channels via direct binding of a cytosolic domain to Orai1. Cell.

[83] Yuan J.P., Zeng W., Dorwart M.R., Choi Y.J., Worley P.F., Muallem S. (2009). SOAR and the polybasic STIM1 domains gate and regulate Orai channels. Nat. Cell Biol..

[84] Kawasaki T., Lange I., Feske S. (2009). A minimal regulatory domain in the C terminus of STIM1 binds to and activates ORAI1 CRAC channels. Biochem. Biophys. Res. Commun..

[85] Hoth M., Penner R. (1993). Calcium release-activated calcium current in rat mast cells. J. Physiol..

[86] Zweifach A., Lewis R.S. (1995). Rapid inactivation of depletion-activated calcium current (ICRAC) due to local calcium feedback. J. Gen. Physiol..

[87] Derler I., Fahrner M., Muik M., Lackner B., Schindl R., Groschner K., Romanin C. (2009). A Ca2(+)release-activated Ca2(+) (CRAC) modulatory domain (CMD) within STIM1 mediates fast Ca2(+)-dependent inactivation of ORAI1 channels. J. Biol. Chem..

[88] Mullins F.M., Lewis R.S. (2016). The inactivation domain of STIM1 is functionally coupled with the Orai1 pore to enable Ca2+-dependent inactivation. J. Gen. Physiol..

[89] Mullins F.M., Yen M., Lewis R.S. (2016). Orai1 pore residues control CRAC channel inactivation independently of calmodulin. J. Gen. Physiol..

[90] Mullins F.M., Park C.Y., Dolmetsch R.E., Lewis R.S. (2009). STIM1 and calmodulin interact with Orai1 to induce Ca2+-dependent inactivation of CRAC channels. Proc. Natl. Acad. Sci. USA.

[91] Choi Y.J., Zhao Y., Bhattacharya M., Stathopulos P.B. (2017). Structural perturbations induced by Asn131 and Asn171 glycosylation converge within the EFSAM core and enhance stromal interaction molecule-1 mediated store operated calcium entry. Biochim. Biophys. Acta Mol. Cell Res..

[92] Gui L., Zhu J., Lu X., Sims S.M., Lu W.Y., Stathopulos P.B., Feng Q. (2018). S-Nitrosylation of STIM1 by Neuronal Nitric Oxide Synthase Inhibits Store-Operated Ca(2+) Entry. J. Mol. Biol..

[93] Zhu J., Lu X., Feng Q., Stathopulos P.B. (2018). A charge-sensing region in the stromal interaction molecule 1 luminal domain confers stabilization-mediated inhibition of SOCE in response to S-nitrosylation. J. Biol. Chem..

[94] Zhu-Mauldin X., Marsh S.A., Zou L., Marchase R.B., Chatham J.C. (2012). Modification of STIM1 by O-linked N-acetylglucosamine (O-GlcNAc) attenuates store-operated calcium entry in neonatal cardiomyocytes. J. Biol. Chem..

[95] Korzeniowski M.K., Popovic M.A., Szentpetery Z., Varnai P., Stojilkovic S.S., Balla T. (2009). Dependence of STIM1/Orai1-mediated calcium entry on plasma membrane phosphoinositides. J. Biol. Chem..

[96] Lopez E., Jardin I., Berna-Erro A., Bermejo N., Salido G.M., Sage S.O., Rosado J.A., Redondo P.C. (2012). STIM1 tyrosine-phosphorylation is required for STIM1-Orai1 association in human platelets. Cell Signal.

[97] Manji S.S., Parker N.J., Williams R.T., van Stekelenburg L., Pearson R.B., Dziadek M., Smith P.J. (2000). STIM1: A novel phosphoprotein located at the cell surface. Biochim. Biophys. Acta.

[98] Pozo-Guisado E., Campbell D.G., Deak M., Alvarez-Barrientos A., Morrice N.A., Alvarez I.S., Alessi D.R., Martin-Romero F.J. (2010). Phosphorylation of STIM1 at ERK1/2 target sites modulates store-operated calcium entry. J. Cell Sci..

[99] Smyth J.T., Beg A.M., Wu S., Putney J.W., Rusan N.M. (2012). Phosphoregulation of STIM1 leads to exclusion of the endoplasmic reticulum from the mitotic spindle. Curr. Biol..

[100] Smyth J.T., Petranka J.G., Boyles R.R., DeHaven W.I., Fukushima M., Johnson K.L., Williams J.G., Putney J.W. (2009). Phosphorylation of STIM1 underlies suppression of store-operated calcium entry during mitosis. Nat. Cell Biol..

[101] Thompson J.L., Lai-Zhao Y., Stathopulos P.B., Grossfield A., Shuttleworth T.J. (2018). Phosphorylation-mediated structural changes within the SOAR domain of stromal interaction molecule 1 enable specific activation of distinct Orai channels. J. Biol. Chem..

[102] Williams R.T., Manji S.S., Parker N.J., Hancock M.S., Van Stekelenburg L., Eid J.P., Senior P.V., Kazenwadel J.S., Shandala T., Saint R. (2001). Identification and characterization of the STIM (stromal interaction molecule) gene family: Coding for a novel class of transmembrane proteins. Biochem. J..

[103] Yazbeck P., Tauseef M., Kruse K., Amin M.R., Sheikh R., Feske S., Komarova Y., Mehta D. (2017). STIM1 Phosphorylation at Y361 Recruits Orai1 to STIM1 Puncta and Induces Ca(2+) Entry. Sci. Rep..

[104] Hawkins B.J., Irrinki K.M., Mallilankaraman K., Lien Y.C., Wang Y., Bhanumathy C.D., Subbiah R., Ritchie M.F., Soboloff J., Baba Y. (2010). S-glutathionylation activates STIM1 and alters mitochondrial homeostasis. J. Cell Biol..

[105] Lupas A., Van Dyke M., Stock J. (1991). Predicting coiled coils from protein sequences. Science.

[106] Cui B., Yang X., Li S., Lin Z., Wang Z., Dong C., Shen Y. (2013). The inhibitory helix controls the intramolecular conformational switching of the C-terminus of STIM1. PLoS ONE.

[107] Stathopulos P.B., Schindl R., Fahrner M., Zheng L., Gasmi-Seabrook G.M., Muik M., Romanin C., Ikura M. (2013). STIM1/Orai1 coiled-coil interplay in the regulation of store-operated calcium entry. Nat. Commun..

[108] Yang X., Jin H., Cai X., Li S., Shen Y. (2012). Structural and mechanistic insights into the activation of Stromal interaction molecule 1 (STIM1). Proc. Natl. Acad. Sci. USA.

[109] Muik M., Fahrner M., Derler I., Schindl R., Bergsmann J., Frischauf I., Groschner K., Romanin C. (2009). A Cytosolic Homomerization and a Modulatory Domain within STIM1 C Terminus Determine Coupling to ORAI1 Channels. J. Biol. Chem..

[110] Covington E.D., Wu M.M., Lewis R.S. (2010). Essential role for the CRAC activation domain in store-dependent oligomerization of STIM1. Mol. Biol. Cell.

[111] Berridge M.J., Irvine R.F. (1984). Inositol trisphosphate, a novel second messenger in cellular signal transduction. Nature.

[112] Streb H., Irvine R.F., Berridge M.J., Schulz I. (1983). Release of Ca2+ from a nonmitochondrial intracellular store in pancreatic acinar cells by inositol-1,4,5-trisphosphate. Nature.

[113] Zheng L., Stathopulos P.B., Schindl R., Li G.Y., Romanin C., Ikura M. (2011). Auto-inhibitory role of the EF-SAM domain of STIM proteins in store-operated calcium entry. Proc. Natl. Acad. Sci. USA.

[114] Ma G., Wei M., He L., Liu C., Wu B., Zhang S.L., Jing J., Liang X., Senes A., Tan P. (2015). Inside-out Ca(2+) signalling prompted by STIM1 conformational switch. Nat. Commun..

[115] Fahrner M., Muik M., Schindl R., Butorac C., Stathopulos P., Zheng L., Jardin I., Ikura M., Romanin C. (2014). A coiled-coil clamp controls both conformation and clustering of stromal interaction molecule 1 (STIM1). J. Biol. Chem..

[116] Calloway N., Owens T., Corwith K., Rodgers W., Holowka D., Baird B. (2011). Stimulated association of STIM1 and Orai1 is regulated by the balance of PtdIns(4,5)P(2) between distinct membrane pools. J. Cell Sci..

[117] Walsh C.M., Chvanov M., Haynes L.P., Petersen O.H., Tepikin A.V., Burgoyne R.D. (2009). Role of phosphoinositides in STIM1 dynamics and store-operated calcium entry. Biochem. J..

[118] Huang G.N., Zeng W., Kim J.Y., Yuan J.P., Han L., Muallem S., Worley P.F. (2006). STIM1 carboxyl-terminus activates native SOC, I(crac) and TRPC1 channels. Nat. Cell Biol..

[119] McNally B.A., Somasundaram A., Jairaman A., Yamashita M., Prakriya M. (2013). The C- and N-terminal STIM1 binding sites on Orai1 are required for both trapping and gating CRAC channels. J. Physiol..

[120] Hou X., Pedi L., Diver M.M., Long S.B. (2012). Crystal structure of the calcium release-activated calcium channel Orai. Science.

[121] Picard C., McCarl C.A., Papolos A., Khalil S., Luthy K., Hivroz C., LeDeist F., Rieux-Laucat F., Rechavi G., Rao A. (2009). STIM1 mutation associated with a syndrome of immunodeficiency and autoimmunity. N. Engl. J. Med..

[122] Byun M., Abhyankar A., Lelarge V., Plancoulaine S., Palanduz A., Telhan L., Boisson B., Picard C., Dewell S., Zhao C. (2010). Whole-exome sequencing-based discovery of STIM1 deficiency in a child with fatal classic Kaposi sarcoma. J. Exp. Med..

[123] Fuchs S., Rensing-Ehl A., Speckmann C., Bengsch B., Schmitt-Graeff A., Bondzio I., Maul-Pavicic A., Bass T., Vraetz T., Strahm B. (2012). Antiviral and regulatory T cell immunity in a patient with stromal interaction molecule 1 deficiency. J. Immunol..

[124] Schaballie H., Rodriguez R., Martin E., Moens L., Frans G., Lenoir C., Dutre J., Canioni D., Bossuyt X., Fischer A. (2015). A novel hypomorphic mutation in STIM1 results in a late-onset immunodeficiency. J. Allergy Clin. Immunol..

[125] Maus M., Jairaman A., Stathopulos P.B., Muik M., Fahrner M., Weidinger C., Benson M., Fuchs S., Ehl S., Romanin C. (2015). Missense mutation in immunodeficient patients shows the multifunctional roles of coiled-coil domain 3 (CC3) in STIM1 activation. Proc. Natl. Acad. Sci. USA.

[126] McCarl C.A., Picard C., Khalil S., Kawasaki T., Rother J., Papolos A., Kutok J., Hivroz C., Ledeist F., Plogmann K. (2009). ORAI1 deficiency and lack of store-operated Ca2+ entry cause immunodeficiency, myopathy, and ectodermal dysplasia. J. Allergy Clin. Immunol..

[127] Chou J., Badran Y.R., Yee C.S.K., Bainter W., Ohsumi T.K., Al-Hammadi S., Pai S.Y., Feske S., Geha R.S. (2015). A novel mutation in ORAI1 presenting with combined immunodeficiency and residual T-cell function. J. Allergy Clin. Immunol..

[128] Feske S. (2019). CRAC channels and disease-From human CRAC channelopathies and animal models to novel drugs. Cell Calcium.

[129] Lacruz R.S., Feske S. (2015). Diseases caused by mutations in ORAI1 and STIM1. Ann. N. Y. Acad. Sci..

[130] Misceo D., Holmgren A., Louch W.E., Holme P.A., Mizobuchi M., Morales R.J., De Paula A.M., Stray-Pedersen A., Lyle R., Dalhus B. (2014). A dominant STIM1 mutation causes Stormorken syndrome. Hum. Mutat..

[131] Bohm J., Chevessier F., Maues De Paula A., Koch C., Attarian S., Feger C., Hantai D., Laforet P., Ghorab K., Vallat J.M. (2013). Constitutive activation of the calcium sensor STIM1 causes tubular-aggregate myopathy. Am. J. Hum. Genet..

[132] Bohm J., Chevessier F., Koch C., Peche G.A., Mora M., Morandi L., Pasanisi B., Moroni I., Tasca G., Fattori F. (2014). Clinical, histological and genetic characterisation of patients with tubular aggregate myopathy caused by mutations in STIM1. J. Med. Genet..

[133] Walter M.C., Rossius M., Zitzelsberger M., Vorgerd M., Muller-Felber W., Ertl-Wagner B., Zhang Y., Brinkmeier H., Senderek J., Schoser B. (2015). 50 years to diagnosis: Autosomal dominant tubular aggregate myopathy caused by a novel STIM1 mutation. Neuromuscul. Disord..

[134] Nesin V., Wiley G., Kousi M., Ong E.C., Lehmann T., Nicholl D.J., Suri M., Shahrizaila N., Katsanis N., Gaffney P.M. (2014). Activating mutations in STIM1 and ORAI1 cause overlapping syndromes of tubular myopathy and congenital miosis. Proc. Natl. Acad. Sci. USA.

[135] Endo Y., Noguchi S., Hara Y., Hayashi Y.K., Motomura K., Miyatake S., Murakami N., Tanaka S., Yamashita S., Kizu R. (2015). Dominant mutations in ORAI1 cause tubular aggregate myopathy with hypocalcemia via constitutive activation of store-operated Ca(2)(+) channels. Hum. Mol. Genet..

[136] Bohm J., Laporte J. (2018). Gain-of-function mutations in STIM1 and ORAI1 causing tubular aggregate myopathy and Stormorken syndrome. Cell Calcium.

[137] Hoth M., Fanger C.M., Lewis R.S. (1997). Mitochondrial regulation of store-operated calcium signaling in T lymphocytes. J. Cell Biol..

[138] Hoth M., Button D.C., Lewis R.S. (2000). Mitochondrial control of calcium-channel gating: A mechanism for sustained signaling and transcriptional activation in T lymphocytes. Proc. Natl. Acad. Sci. USA.

[139] Quintana A., Schwarz E.C., Schwindling C., Lipp P., Kaestner L., Hoth M. (2006). Sustained activity of calcium release-activated calcium channels requires translocation of mitochondria to the plasma membrane. J. Biol. Chem..

[140] Naghdi S., Waldeck-Weiermair M., Fertschai I., Poteser M., Graier W.F., Malli R. (2010). Mitochondrial Ca2+ uptake and not mitochondrial motility is required for STIM1-Orai1-dependent store-operated Ca2+ entry. J. Cell Sci..

[141] Gilabert J.A., Parekh A.B. (2000). Respiring mitochondria determine the pattern of activation and inactivation of the store-operated Ca(2+) current I(CRAC). EMBO J..

[142] Samanta K., Douglas S., Parekh A.B. (2014). Mitochondrial calcium uniporter MCU supports cytoplasmic Ca2+ oscillations, store-operated Ca2+ entry and Ca2+-dependent gene expression in response to receptor stimulation. PLoS ONE.

[143] Kostic M., Sekler I. (2019). Functional properties and mode of regulation of the mitochondrial Na(+)/Ca(2+) exchanger, NCLX. Semin Cell Dev. Biol..

[144] Ben-Kasus Nissim T., Zhang X., Elazar A., Roy S., Stolwijk J.A., Zhou Y., Motiani R.K., Gueguinou M., Hempel N., Hershfinkel M. (2017). Mitochondria control store-operated Ca(2+) entry through Na(+) and redox signals. EMBO J..

[145] Villalobos C., Gutierrez L.G., Hernandez-Morales M., Del Bosque D., Nunez L. (2018). Mitochondrial control of store-operated Ca(2+) channels in cancer: Pharmacological implications. Pharmacol. Res..

[146] Marchi S., Pinton P. (2014). The mitochondrial calcium uniporter complex: Molecular components, structure and physiopathological implications. J. Physiol..

[147] Giacomello M., Drago I., Pizzo P., Pozzan T. (2007). Mitochondrial Ca2+ as a key regulator of cell life and death. Cell Death Differ..

[148] Shoshan-Barmatz V., De S. (2017). Mitochondrial VDAC, the Na(+)/Ca(2+) Exchanger, and the Ca(2+) Uniporter in Ca(2+) Dynamics and Signaling. Adv. Exp. Med. Biol..

[149] Mallilankaraman K., Doonan P., Cardenas C., Chandramoorthy H.C., Muller M., Miller R., Hoffman N.E., Gandhirajan R.K., Molgo J., Birnbaum M.J. (2012). MICU1 is an essential gatekeeper for MCU-mediated mitochondrial Ca(2+) uptake that regulates cell survival. Cell.

[150] Rizzuto R., Pozzan T. (2006). Microdomains of intracellular Ca2+: Molecular determinants and functional consequences. Physiol. Rev..

[151] Baradaran R., Wang C., Siliciano A.F., Long S.B. (2018). Cryo-EM structures of fungal and metazoan mitochondrial calcium uniporters. Nature.

[152] Fan C., Fan M., Orlando B.J., Fastman N.M., Zhang J., Xu Y., Chambers M.G., Xu X., Perry K., Liao M. (2018). X-ray and cryo-EM structures of the mitochondrial calcium uniporter. Nature.

[153] Nguyen N.X., Armache J.P., Lee C., Yang Y., Zeng W., Mootha V.K., Cheng Y., Bai X.C., Jiang Y. (2018). Cryo-EM structure of a fungal mitochondrial calcium uniporter. Nature.

[154] Wang Y., Nguyen N.X., She J., Zeng W., Yang Y., Bai X.C., Jiang Y. (2019). Structural Mechanism of EMRE-Dependent Gating of the Human Mitochondrial Calcium Uniporter. Cell.

[155] Yoo J., Wu M., Yin Y., Herzik M.A., Lander G.C., Lee S.Y. (2018). Cryo-EM structure of a mitochondrial calcium uniporter. Science.

[156] Perocchi F., Gohil V.M., Girgis H.S., Bao X.R., McCombs J.E., Palmer A.E., Mootha V.K. (2010). MICU1 encodes a mitochondrial EF hand protein required for Ca(2+) uptake. Nature.

[157] Plovanich M., Bogorad R.L., Sancak Y., Kamer K.J., Strittmatter L., Li A.A., Girgis H.S., Kuchimanchi S., De Groot J., Speciner L. (2013). MICU2, a paralog of MICU1, resides within the mitochondrial uniporter complex to regulate calcium handling. PLoS ONE.

[158] Sancak Y., Markhard A.L., Kitami T., Kovacs-Bogdan E., Kamer K.J., Udeshi N.D., Carr S.A., Chaudhuri D., Clapham D.E., Li A.A. (2013). EMRE is an essential component of the mitochondrial calcium uniporter complex. Science.

[159] Raffaello A., De Stefani D., Sabbadin D., Teardo E., Merli G., Picard A., Checchetto V., Moro S., Szabo I., Rizzuto R. (2013). The mitochondrial calcium uniporter is a multimer that can include a dominant-negative pore-forming subunit. EMBO J..

[160] Mallilankaraman K., Cardenas C., Doonan P.J., Chandramoorthy H.C., Irrinki K.M., Golenar T., Csordas G., Madireddi P., Yang J., Muller M. (2012). MCUR1 is an essential component of mitochondrial Ca2+ uptake that regulates cellular metabolism. Nat. Cell Biol..

[161] Martell J.D., Deerinck T.J., Sancak Y., Poulos T.L., Mootha V.K., Sosinsky G.E., Ellisman M.H., Ting A.Y. (2012). Engineered ascorbate peroxidase as a genetically encoded reporter for electron microscopy. Nat. Biotechnol..

[162] Oxenoid K., Dong Y., Cao C., Cui T., Sancak Y., Markhard A.L., Grabarek Z., Kong L., Liu Z., Ouyang B. (2016). Architecture of the mitochondrial calcium uniporter. Nature.

[163] Lee Y., Min C.K., Kim T.G., Song H.K., Lim Y., Kim D., Shin K., Kang M., Kang J.Y., Youn H.S. (2015). Structure and function of the N-terminal domain of the human mitochondrial calcium uniporter. EMBO Rep..

[164] Lee S.K., Shanmughapriya S., Mok M.C.Y., Dong Z., Tomar D., Carvalho E., Rajan S., Junop M.S., Madesh M., Stathopulos P.B. (2016). Structural Insights into Mitochondrial Calcium Uniporter Regulation by Divalent Cations. Cell Chem. Biol..

[165] Dong Z., Shanmughapriya S., Tomar D., Siddiqui N., Lynch S., Nemani N., Breves S.L., Zhang X., Tripathi A., Palaniappan P. (2017). Mitochondrial Ca(2+) Uniporter Is a Mitochondrial Luminal Redox Sensor that Augments MCU Channel Activity. Mol. Cell.

[166] Vais H., Mallilankaraman K., Mak D.D., Hoff H., Payne R., Tanis J.E., Foskett J.K. (2016). EMRE Is a Matrix Ca(2+) Sensor that Governs Gatekeeping of the Mitochondrial Ca(2+) Uniporter. Cell Rep..

[167] Tsai M.F., Phillips C.B., Ranaghan M., Tsai C.W., Wu Y., Willliams C., Miller C. (2016). Dual functions of a small regulatory subunit in the mitochondrial calcium uniporter complex. Elife.

[168] Hung V., Zou P., Rhee H.W., Udeshi N.D., Cracan V., Svinkina T., Carr S.A., Mootha V.K., Ting A.Y. (2014). Proteomic mapping of the human mitochondrial intermembrane space in live cells via ratiometric APEX tagging. Mol. Cell.

[169] Csordas G., Golenar T., Seifert E.L., Kamer K.J., Sancak Y., Perocchi F., Moffat C., Weaver D., de la Fuente Perez S., Bogorad R. (2013). MICU1 controls both the threshold and cooperative activation of the mitochondrial Ca(2)(+) uniporter. Cell Metab..

[170] Patron M., Checchetto V., Raffaello A., Teardo E., Vecellio Reane D., Mantoan M., Granatiero V., Szabo I., De Stefani D., Rizzuto R. (2014). MICU1 and MICU2 finely tune the mitochondrial Ca2+ uniporter by exerting opposite effects on MCU activity. Mol. Cell.

[171] Patron M., Granatiero V., Espino J., Rizzuto R., De Stefani D. (2019). MICU3 is a tissue-specific enhancer of mitochondrial calcium uptake. Cell Death Differ..

[172] Kamer K.J., Grabarek Z., Mootha V.K. (2017). High-affinity cooperative Ca(2+) binding by MICU1-MICU2 serves as an on-off switch for the uniporter. EMBO Rep..

[173] Kamer K.J., Jiang W., Kaushik V.K., Mootha V.K., Grabarek Z. (2019). Crystal structure of MICU2 and comparison with MICU1 reveal insights into the uniporter gating mechanism. Proc. Natl. Acad. Sci. USA.

[174] Kamer K.J., Mootha V.K. (2014). MICU1 and MICU2 play nonredundant roles in the regulation of the mitochondrial calcium uniporter. EMBO Rep..

[175] Xing Y., Wang M., Wang J., Nie Z., Wu G., Yang X., Shen Y. (2019). Dimerization of MICU Proteins Controls Ca(2+) Influx through the Mitochondrial Ca(2+) Uniporter. Cell Rep..

[176] Park J., Lee Y., Park T., Kang J.Y., Mun S.A., Jin M., Yang J., Eom S.H. (2020). Structure of the MICU1-MICU2 heterodimer provides insights into the gatekeeping threshold shift. IUCrJ.

[177] Wang L., Yang X., Li S., Wang Z., Liu Y., Feng J., Zhu Y., Shen Y. (2014). Structural and mechanistic insights into MICU1 regulation of mitochondrial calcium uptake. EMBO J..

[178] Wu W., Shen Q., Lei Z., Qiu Z., Li D., Pei H., Zheng J., Jia Z. (2019). The crystal structure of MICU2 provides insight into Ca(2+) binding and MICU1-MICU2 heterodimer formation. EMBO Rep..

[179] Zhuo W., Zhou H., Guo R., Yi J., Sui Y., Zhang L., Zeng W., Wang P., Yang M. (2020). Structure of intact human MCU supercomplex with the auxiliary MICU subunits. bioRxiv.

[180] Lambert J.P., Luongo T.S., Tomar D., Jadiya P., Gao E., Zhang X., Lucchese A.M., Kolmetzky D.W., Shah N.S., Elrod J.W. (2019). MCUB Regulates the Molecular Composition of the Mitochondrial Calcium Uniporter Channel to Limit Mitochondrial Calcium Overload During Stress. Circulation.

[181] Tomar D., Dong Z., Shanmughapriya S., Koch D.A., Thomas T., Hoffman N.E., Timbalia S.A., Goldman S.J., Breves S.L., Corbally D.P. (2016). MCUR1 Is a Scaffold Factor for the MCU Complex Function and Promotes Mitochondrial Bioenergetics. Cell Rep..

[182] Vais H., Tanis J.E., Muller M., Payne R., Mallilankaraman K., Foskett J.K. (2015). MCUR1, CCDC90A, Is a Regulator of the Mitochondrial Calcium Uniporter. Cell Metab..

[183] Rizzuto R., De Stefani D., Raffaello A., Mammucari C. (2012). Mitochondria as sensors and regulators of calcium signalling. Nat. Rev. Mol. Cell Biol..

[184] Sileikyte J., Forte M. (2019). The Mitochondrial Permeability Transition in Mitochondrial Disorders. Oxid Med. Cell Longev..

[185] Kwong J.Q., Molkentin J.D. (2015). Physiological and pathological roles of the mitochondrial permeability transition pore in the heart. Cell Metab..

[186] Zoratti M., Szabo I. (1995). The mitochondrial permeability transition. Biochim. Biophys. Acta.

[187] Bhosale G., Sharpe J.A., Koh A., Kouli A., Szabadkai G., Duchen M.R. (2017). Pathological consequences of MICU1 mutations on mitochondrial calcium signalling and bioenergetics. Biochim. Biophys. Acta Mol. Cell Res..

[188] Halestrap A.P., Richardson A.P. (2015). The mitochondrial permeability transition: A current perspective on its identity and role in ischaemia/reperfusion injury. J. Mol. Cell Cardiol..

[189] Liao Y., Dong Y., Cheng J. (2017). The Function of the Mitochondrial Calcium Uniporter in Neurodegenerative Disorders. Int. J. Mol. Sci..

[190] Tarasov A.I., Semplici F., Ravier M.A., Bellomo E.A., Pullen T.J., Gilon P., Sekler I., Rizzuto R., Rutter G.A. (2012). The mitochondrial Ca2+ uniporter MCU is essential for glucose-induced ATP increases in pancreatic beta-cells. PLoS ONE.

[191] Vultur A., Gibhardt C.S., Stanisz H., Bogeski I. (2018). The role of the mitochondrial calcium uniporter (MCU) complex in cancer. Pflugers Arch..

[192] Debattisti V., Horn A., Singh R., Seifert E.L., Hogarth M.W., Mazala D.A., Huang K.T., Horvath R., Jaiswal J.K., Hajnoczky G. (2019). Dysregulation of Mitochondrial Ca(2+) Uptake and Sarcolemma Repair Underlie Muscle Weakness and Wasting in Patients and Mice Lacking MICU1. Cell Rep..

[193] Logan C.V., Szabadkai G., Sharpe J.A., Parry D.A., Torelli S., Childs A.M., Kriek M., Phadke R., Johnson C.A., Roberts N.Y. (2014). Loss-of-function mutations in MICU1 cause a brain and muscle disorder linked to primary alterations in mitochondrial calcium signaling. Nat. Genet..

[194] Lewis-Smith D., Kamer K.J., Griffin H., Childs A.M., Pysden K., Titov D., Duff J., Pyle A., Taylor R.W., Yu-Wai-Man P. (2016). Homozygous deletion in MICU1 presenting with fatigue and lethargy in childhood. Neurol. Genet..

[195] Musa S., Eyaid W., Kamer K., Ali R., Al-Mureikhi M., Shahbeck N., Al Mesaifri F., Makhseed N., Mohamed Z., AlShehhi W.A. (2019). A Middle Eastern Founder Mutation Expands the Genotypic and Phenotypic Spectrum of Mitochondrial MICU1 Deficiency: A Report of 13 Patients. JIMD Rep..

[196] Shamseldin H.E., Alasmari A., Salih M.A., Samman M.M., Mian S.A., Alshidi T., Ibrahim N., Hashem M., Faqeih E., Al-Mohanna F. (2017). A null mutation in MICU2 causes abnormal mitochondrial calcium homeostasis and a severe neurodevelopmental disorder. Brain.

[197] Gordienko D.V., Greenwood I.A., Bolton T.B. (2001). Direct visualization of sarcoplasmic reticulum regions discharging Ca(2+)sparks in vascular myocytes. Cell Calcium.

[198] Hajnoczky G., Hager R., Thomas A.P. (1999). Mitochondria suppress local feedback activation of inositol 1,4, 5-trisphosphate receptors by Ca2+. J. Biol. Chem..

[199] Marchant J.S., Ramos V., Parker I. (2002). Structural and functional relationships between Ca2+ puffs and mitochondria in Xenopus oocytes. Am. J. Physiol. Cell Physiol..

[200] Pacher P., Thomas A.P., Hajnoczky G. (2002). Ca2+ marks: Miniature calcium signals in single mitochondria driven by ryanodine receptors. Proc. Natl. Acad. Sci. USA.

[201] Antony A.N., Paillard M., Moffat C., Juskeviciute E., Correnti J., Bolon B., Rubin E., Csordas G., Seifert E.L., Hoek J.B. (2016). MICU1 regulation of mitochondrial Ca(2+) uptake dictates survival and tissue regeneration. Nat. Commun..

[202] Austin S., Nowikovsky K. (2019). LETM1: Essential for Mitochondrial Biology and Cation Homeostasis?. Trends Biochem. Sci..

[203] Doonan P.J., Chandramoorthy H.C., Hoffman N.E., Zhang X., Cardenas C., Shanmughapriya S., Rajan S., Vallem S., Chen X., Foskett J.K. (2014). LETM1-dependent mitochondrial Ca2+ flux modulates cellular bioenergetics and proliferation. FASEB J..

[204] Jiang D., Zhao L., Clish C.B., Clapham D.E. (2013). Letm1, the mitochondrial Ca2+/H+ antiporter, is essential for normal glucose metabolism and alters brain function in Wolf-Hirschhorn syndrome. Proc. Natl. Acad. Sci. USA.

[205] Nowikovsky K., Reipert S., Devenish R.J., Schweyen R.J. (2007). Mdm38 protein depletion causes loss of mitochondrial K+/H+ exchange activity, osmotic swelling and mitophagy. Cell Death Differ..

[206] Shao J., Fu Z., Ji Y., Guan X., Guo S., Ding Z., Yang X., Cong Y., Shen Y. (2016). Leucine zipper-EF-hand containing transmembrane protein 1 (LETM1) forms a Ca(2+)/H(+) antiporter. Sci. Rep..

[207] Tamai S., Iida H., Yokota S., Sayano T., Kiguchiya S., Ishihara N., Hayashi J., Mihara K., Oka T. (2008). Characterization of the mitochondrial protein LETM1, which maintains the mitochondrial tubular shapes and interacts with the AAA-ATPase BCS1L. J. Cell Sci..

[208] Tsai M.F., Jiang D., Zhao L., Clapham D., Miller C. (2014). Functional reconstitution of the mitochondrial Ca2+/H+ antiporter Letm1. J. Gen. Physiol..

[209] Endele S., Fuhry M., Pak S.J., Zabel B.U., Winterpacht A. (1999). LETM1, a novel gene encoding a putative EF-hand Ca(2+)-binding protein, flanks the Wolf-Hirschhorn syndrome (WHS) critical region and is deleted in most WHS patients. Genomics.

[210] Rutherford E.L., Lowery L.A. (2016). Exploring the developmental mechanisms underlying Wolf-Hirschhorn Syndrome: Evidence for defects in neural crest cell migration. Dev. Biol..

[211] Blomen V.A., Majek P., Jae L.T., Bigenzahn J.W., Nieuwenhuis J., Staring J., Sacco R., van Diemen F.R., Olk N., Stukalov A. (2015). Gene essentiality and synthetic lethality in haploid human cells. Science.

[212] Wang T., Birsoy K., Hughes N.W., Krupczak K.M., Post Y., Wei J.J., Lander E.S., Sabatini D.M. (2015). Identification and characterization of essential genes in the human genome. Science.

[213] Collins T.J., Lipp P., Berridge M.J., Bootman M.D. (2001). Mitochondrial Ca(2+) uptake depends on the spatial and temporal profile of cytosolic Ca(2+) signals. J. Biol. Chem..

[214] Santo-Domingo J., Demaurex N. (2010). Calcium uptake mechanisms of mitochondria. Biochim. Biophys. Acta.

[215] Santo-Domingo J., Demaurex N. (2012). Perspectives on: SGP symposium on mitochondrial physiology and medicine: The renaissance of mitochondrial pH. J. Gen. Physiol..

[216] Aral C., Demirkesen S., Bircan R., Yasar Sirin D. (2020). Melatonin reverses the oxidative stress and mitochondrial dysfunction caused by LETM1 silencing. Cell Biol. Int..

[217] Huang E., Qu D., Huang T., Rizzi N., Boonying W., Krolak D., Ciana P., Woulfe J., Klein C., Slack R.S. (2017). PINK1-mediated phosphorylation of LETM1 regulates mitochondrial calcium transport and protects neurons against mitochondrial stress. Nat. Commun..

[218] Yoo C.M., Rhee H.W. (2020). APEX, a Master Key To Resolve Membrane Topology in Live Cells. Biochemistry.

[219] Lupo D., Vollmer C., Deckers M., Mick D.U., Tews I., Sinning I., Rehling P. (2011). Mdm38 is a 14-3-3-like receptor and associates with the protein synthesis machinery at the inner mitochondrial membrane. Traffic.

[220] Nakamura S., Matsui A., Akabane S., Tamura Y., Hatano A., Miyano Y., Omote H., Kajikawa M., Maenaka K., Moriyama Y. (2020). The mitochondrial inner membrane protein LETM1 modulates cristae organization through its LETM domain. Commun. Biol..

[221] Huang B., Zhang J., Zhang X., Huang C., Hu G., Li S., Xie T., Liu M., Xu Y. (2017). Suppression of LETM1 by siRNA inhibits cell proliferation and invasion of bladder cancer cells. Oncol. Rep..

[222] Piao L., Li Y., Kim S.J., Byun H.S., Huang S.M., Hwang S.K., Yang K.J., Park K.A., Won M., Hong J. (2009). Association of LETM1 and MRPL36 contributes to the regulation of mitochondrial ATP production and necrotic cell death. Cancer Res..

[223] Piao L., Li Y., Kim S.J., Sohn K.C., Yang K.J., Park K.A., Byun H.S., Won M., Hong J., Hur G.M. (2009). Regulation of OPA1-mediated mitochondrial fusion by leucine zipper/EF-hand-containing transmembrane protein-1 plays a role in apoptosis. Cell Signal..

[224] Yang Z., Ni W., Cui C., Qi W., Piao L., Xuan Y. (2018). Identification of LETM1 as a marker of cancer stem-like cells and predictor of poor prognosis in esophageal squamous cell carcinoma. Hum. Pathol..

[225] Hou X., Burstein S.R., Long S.B. (2018). Structures reveal opening of the store-operated calcium channel Orai. Elife.

[226] Liu X., Wu G., Yu Y., Chen X., Ji R., Lu J., Li X., Zhang X., Yang X., Shen Y. (2019). Molecular understanding of calcium permeation through the open Orai channel. PLoS Biol..

[227] Yuan Y., Cao C., Wen M., Li M., Dong Y., Wu L., Wu J., Cui T., Li D., Chou J.J. (2020). Structural Characterization of the N-Terminal Domain of the Dictyostelium discoideum Mitochondrial Calcium Uniporter. ACS Omega..

[228] Adlakha J., Karamichali I., Sangwallek J., Deiss S., Bar K., Coles M., Hartmann M.D., Lupas A.N., Hernandez Alvarez B. (2019). Characterization of MCU-Binding Proteins MCUR1 and CCDC90B-Representatives of a Protein Family Conserved in Prokaryotes and Eukaryotic Organelles. Structure.

[229] Bosanac I., Alattia J.R., Mal T.K., Chan J., Talarico S., Tong F.K., Tong K.I., Yoshikawa F., Furuichi T., Iwai M. (2002). Structure of the inositol 1,4,5-trisphosphate receptor binding core in complex with its ligand. Nature.

[230] Bosanac I., Yamazaki H., Matsu-Ura T., Michikawa T., Mikoshiba K., Ikura M. (2005). Crystal structure of the ligand binding suppressor domain of type 1 inositol 1,4,5-trisphosphate receptor. Mol. Cell.

[231] Amador F.J., Kimlicka L., Stathopulos P.B., Gasmi-Seabrook G.M., Maclennan D.H., Van Petegem F., Ikura M. (2013). Type 2 ryanodine receptor domain A contains a unique and dynamic alpha-helix that transitions to a beta-strand in a mutant linked with a heritable cardiomyopathy. J. Mol. Biol..

[232] Amador F.J., Liu S., Ishiyama N., Plevin M.J., Wilson A., MacLennan D.H., Ikura M. (2009). Crystal structure of type I ryanodine receptor amino-terminal beta-trefoil domain reveals a disease-associated mutation “hot spot” loop. Proc. Natl. Acad. Sci. USA.

[233] Seo M.D., Velamakanni S., Ishiyama N., Stathopulos P.B., Rossi A.M., Khan S.A., Dale P., Li C., Ames J.B., Ikura M. (2012). Structural and functional conservation of key domains in InsP3 and ryanodine receptors. Nature.

[234] Tung C.C., Lobo P.A., Kimlicka L., Van Petegem F. (2010). The amino-terminal disease hotspot of ryanodine receptors forms a cytoplasmic vestibule. Nature.

[235] des Georges A., Clarke O.B., Zalk R., Yuan Q., Condon K.J., Grassucci R.A., Hendrickson W.A., Marks A.R., Frank J. (2016). Structural Basis for Gating and Activation of RyR1. Cell.

[236] Peng W., Shen H., Wu J., Guo W., Pan X., Wang R., Chen S.R., Yan N. (2016). Structural basis for the gating mechanism of the type 2 ryanodine receptor RyR2. Science.

[237] Fan G., Baker M.L., Wang Z., Baker M.R., Sinyagovskiy P.A., Chiu W., Ludtke S.J., Serysheva I.I. (2015). Gating machinery of InsP3R channels revealed by electron cryomicroscopy. Nature.

[238] Liao J., Li H., Zeng W., Sauer D.B., Belmares R., Jiang Y. (2012). Structural insight into the ion-exchange mechanism of the sodium/calcium exchanger. Science.

[239] Toyoshima C., Nomura H., Tsuda T. (2004). Lumenal gating mechanism revealed in calcium pump crystal structures with phosphate analogues. Nature.

[240] Tsunekawa N., Ogawa H., Tsueda J., Akiba T., Toyoshima C. (2018). Mechanism of the E2 to E1 transition in Ca(2+) pump revealed by crystal structures of gating residue mutants. Proc. Natl. Acad. Sci. USA.

[241] Zhao Y., Huang G., Wu J., Wu Q., Gao S., Yan Z., Lei J., Yan N. (2019). Molecular Basis for Ligand Modulation of a Mammalian Voltage-Gated Ca(2+) Channel. Cell.

[242] Tang L., Gamal El-Din T.M., Swanson T.M., Pryde D.C., Scheuer T., Zheng N., Catterall W.A. (2016). Structural basis for inhibition of a voltage-gated Ca(2+) channel by Ca(2+) antagonist drugs. Nature.

[243] Imbrici P., Nicolotti O., Leonetti F., Conte D., Liantonio A. (2018). Ion Channels in Drug Discovery and Safety Pharmacology. Methods Mol. Biol..

[244] Santos R., Ursu O., Gaulton A., Bento A.P., Donadi R.S., Bologa C.G., Karlsson A., Al-Lazikani B., Hersey A., Oprea T.I. (2017). A comprehensive map of molecular drug targets. Nat. Rev. Drug Discov..

[245] Wulff H., Christophersen P., Colussi P., Chandy K.G., Yarov-Yarovoy V. (2019). Antibodies and venom peptides: New modalities for ion channels. Nat. Rev. Drug Discov..

[246] Faulds D., Goa K.L., Benfield P. (1993). Cyclosporin. A review of its pharmacodynamic and pharmacokinetic properties, and therapeutic use in immunoregulatory disorders. Drugs.

[247] Flores C., Fouquet G., Moura I.C., Maciel T.T., Hermine O. (2019). Lessons to Learn From Low-Dose Cyclosporin-A: A New Approach for Unexpected Clinical Applications. Front. Immunol..

[248] Garcia-Rivas Gde J., Carvajal K., Correa F., Zazueta C. (2006). Ru360, a specific mitochondrial calcium uptake inhibitor, improves cardiac post-ischaemic functional recovery in rats in vivo. Br. J. Pharmacol..

[249] Zhang S.Z., Gao Q., Cao C.M., Bruce I.C., Xia Q. (2006). Involvement of the mitochondrial calcium uniporter in cardioprotection by ischemic preconditioning. Life Sci..

[250] de Jesus Garcia-Rivas G., Guerrero-Hernandez A., Guerrero-Serna G., Rodriguez-Zavala J.S., Zazueta C. (2005). Inhibition of the mitochondrial calcium uniporter by the oxo-bridged dinuclear ruthenium amine complex (Ru360) prevents from irreversible injury in postischemic rat heart. FEBS J..

[251] Woods J.J., Nemani N., Shanmughapriya S., Kumar A., Zhang M., Nathan S.R., Thomas M., Carvalho E., Ramachandran K., Srikantan S. (2019). A Selective and Cell-Permeable Mitochondrial Calcium Uniporter (MCU) Inhibitor Preserves Mitochondrial Bioenergetics after Hypoxia/Reoxygenation Injury. ACS Cent. Sci..

[252] Arduino D.M., Wettmarshausen J., Vais H., Navas-Navarro P., Cheng Y., Leimpek A., Ma Z., Delrio-Lorenzo A., Giordano A., Garcia-Perez C. (2017). Systematic Identification of MCU Modulators by Orthogonal Interspecies Chemical Screening. Mol. Cell.

[253] Kon N., Murakoshi M., Isobe A., Kagechika K., Miyoshi N., Nagayama T. (2017). DS16570511 is a small-molecule inhibitor of the mitochondrial calcium uniporter. Cell Death Discov..

[254] Di Marco G., Vallese F., Jourde B., Bergsdorf C., Sturlese M., De Mario A., Techer-Etienne V., Haasen D., Oberhauser B., Schleeger S. (2020). A High-Throughput Screening Identifies MICU1 Targeting Compounds. Cell Rep..

[255] Goto J., Suzuki A.Z., Ozaki S., Matsumoto N., Nakamura T., Ebisui E., Fleig A., Penner R., Mikoshiba K. (2010). Two novel 2-aminoethyl diphenylborinate (2-APB) analogues differentially activate and inhibit store-operated Ca(2+) entry via STIM proteins. Cell Calcium.

[256] Kim K.D., Srikanth S., Tan Y.V., Yee M.K., Jew M., Damoiseaux R., Jung M.E., Shimizu S., An D.S., Ribalet B. (2014). Calcium signaling via Orai1 is essential for induction of the nuclear orphan receptor pathway to drive Th17 differentiation. J. Immunol..

[257] Stauderman K.A. (2018). CRAC channels as targets for drug discovery and development. Cell Calcium.

[258] Zhang H.Z., Xu X.L., Chen H.Y., Ali S., Wang D., Yu J.W., Xu T., Nan F.J. (2015). Discovery and structural optimization of 1-phenyl-3-(1-phenylethyl)urea derivatives as novel inhibitors of CRAC channel. Acta Pharmacol. Sin..

[259] Sadaghiani A.M., Lee S.M., Odegaard J.I., Leveson-Gower D.B., McPherson O.M., Novick P., Kim M.R., Koehler A.N., Negrin R., Dolmetsch R.E. (2014). Identification of Orai1 channel inhibitors by using minimal functional domains to screen small molecule microarrays. Chem. Biol..

[260] Azimi I., Flanagan J.U., Stevenson R.J., Inserra M., Vetter I., Monteith G.R., Denny W.A. (2017). Evaluation of known and novel inhibitors of Orai1-mediated store operated Ca(2+) entry in MDA-MB-231 breast cancer cells using a Fluorescence Imaging Plate Reader assay. Bioorg. Med. Chem..

[261] Rahman S., Rahman T. (2017). Unveiling some FDA-approved drugs as inhibitors of the store-operated Ca(2+) entry pathway. Sci. Rep..

[262] Sterling T., Irwin J.J. (2015). ZINC 15--Ligand Discovery for Everyone. J. Chem. Inf. Model..

[263] Trott O., Olson A.J. (2010). AutoDock Vina: Improving the speed and accuracy of docking with a new scoring function, efficient optimization, and multithreading. J. Comput. Chem..

[264] Andricopulo A.D., Montanari C.A. (2005). Structure-activity relationships for the design of small-molecule inhibitors. Mini. Rev. Med. Chem..

[265] Gruber S.J., Cornea R.L., Li J., Peterson K.C., Schaaf T.M., Gillispie G.D., Dahl R., Zsebo K.M., Robia S.L., Thomas D.D. (2014). Discovery of enzyme modulators via high-throughput time-resolved FRET in living cells. J. Biomol. Screen..

[266] Schaaf T.M., Peterson K.C., Grant B.D., Bawaskar P., Yuen S., Li J., Muretta J.M., Gillispie G.D., Thomas D.D. (2017). High-Throughput Spectral and Lifetime-Based FRET Screening in Living Cells to Identify Small-Molecule Effectors of SERCA. SLAS Discov..

[267] Rebbeck R.T., Essawy M.M., Nitu F.R., Grant B.D., Gillispie G.D., Thomas D.D., Bers D.M., Cornea R.L. (2017). High-Throughput Screens to Discover Small-Molecule Modulators of Ryanodine Receptor Calcium Release Channels. SLAS Discov..

[268] Rebbeck R.T., Singh D.P., Janicek K.A., Bers D.M., Thomas D.D., Launikonis B.S., Cornea R.L. (2020). RyR1-targeted drug discovery pipeline integrating FRET-based high-throughput screening and human myofiber dynamic Ca(2+) assays. Sci. Rep..

